# Association of Baseline Inflammation With Effectiveness of Nutritional Support Among Patients With Disease-Related Malnutrition: A Secondary Analysis of a Randomized Clinical Trial

**DOI:** 10.1001/jamanetworkopen.2020.0663

**Published:** 2020-03-10

**Authors:** Meret Merker, Martina Felder, Louise Gueissaz, Rebekka Bolliger, Pascal Tribolet, Nina Kägi-Braun, Filomena Gomes, Claus Hoess, Vojtech Pavlicek, Stefan Bilz, Sarah Sigrist, Michael Brändle, Christoph Henzen, Robert Thomann, Jonas Rutishauser, Drahomir Aujesky, Nicolas Rodondi, Jaques Donzé, Zeno Stanga, Beat Mueller, Philipp Schuetz

**Affiliations:** 1 Medical University Department, University of Basel, Kantonsspital Aarau, Aarau, Switzerland; 2 University of Basel, Basel, Switzerland; 3 Department of Health Professions, Bern University of Applied Sciences, Bern, Switzerland; 4 Internal Medicine, Kantonsspital Muensterlingen, Muensterlingen, Switzerland; 5 New York Academy of Sciences, New York; 6 Internal Medicine and Endocrinology/Diabetes, Kantonsspital St Gallen, St Gallen, Switzerland; 7 Internal Medicine, Kantonsspital Luzern, Luzern, Switzerland; 8 Internal Medicine, Kantonsspital Solothurn, Solothurn, Switzerland; 9 Internal Medicine, Kantonsspital Baselland, Baselland, Switzerland; 10 Department of General Internal Medicine, Inselspital, Bern University Hospital, University of Bern, Bern, Switzerland; 11 Institute of Primary Health Care (BIHAM), University of Bern, Bern, Switzerland; 12 Division of General Internal Medicine, Brigham and Women’s Hospital, Boston, Massachusetts; 13 Division of Diabetology, Endocrinology, Nutritional Medicine, and Metabolism, Bern University Hospital, Inselspital, University of Bern, Bern, Switzerland

## Abstract

**Importance:**

Inflammation is a key driver of malnutrition during illness and is often accompanied by metabolic effects, including insulin resistance and reduction of appetite. However, it still remains unclear if inflammation influences the response to nutritional support among patients with disease-related malnutrition.

**Objective:**

To examine whether patients’ baseline inflammatory status is associated with the effect of nutritional support on 30-day mortality.

**Design, Setting, and Participants:**

This is a secondary analysis of the Effect of Early Nutritional Support on Frailty, Functional Outcomes, and Recovery of Malnourished Medical Inpatients Trial (EFFORT), a randomized clinical trial conducted in 8 Swiss hospitals from April 2014 to February 2018. A total of 1950 participants who had C-reactive protein measurements at the time of admission were included in this secondary analysis. Data analysis was conducted between June and July 2019.

**Interventions:**

Hospitalized patients at risk for malnutrition were randomly assigned to receive protocol-guided individualized nutritional support to reach protein and energy goals (intervention group) or standard hospital food (control group).

**Main Outcomes and Measures:**

The primary end point was 30-day mortality. Based on C-reactive protein levels at admission, patients were stratified into groups with low, moderate, or high inflammation (<10 mg/L, 10-100 mg/L, and >100 mg/L, respectively).

**Results:**

A total of 1950 patients (median [interquartile range] age, 75 [65-83] years; 1025 [52.6%] men) were included; 533 (27.3%) had low levels of inflammation, 894 (45.9%) had moderate levels of inflammation, and 523 (26.8%) had high levels of inflammation. Compared with the control group, patients receiving nutritional support showed a significant reduction in 30-day mortality, regardless of C-reactive protein level (adjusted odds ratio, 0.61; 95% CI, 0.43-0.86; *P* = .005). In the subgroup of patients with high inflammation, there was no beneficial effect of nutritional support (adjusted odds ratio, 1.32; 95% CI, 0.70-2.50; *P* = .39), providing evidence that inflammation has a significant modifying association (*P* for interaction = .005).

**Conclusions and Relevance:**

Based on this secondary analysis of a multicenter randomized trial, a patient’s admission inflammatory status was associated with their response to nutritional support. If validated in future clinical trials, nutritional support may need to be individualized based on a patient’s initial presentation and markers of inflammation. These results may also help to explain some of the heterogeneity in treatment effects of nutrition seen in previous critical care trials.

**Trial Registration:**

ClinicalTrials.gov Identifier: NCT02517476

## Key Points

**Question:**

Does nutritional support have a similar effect on 30-day mortality among patients with high inflammation compared with patients with low or moderate inflammation?

**Findings:**

In this secondary analysis of a Swiss multicenter trial, including 1950 patients at risk of malnutrition, patients with high levels of inflammation based on their levels of C-reactive protein at admission were not associated with a beneficial effect of nutritional support on 30-day mortality compared with the overall population, suggesting that inflammation has a significant modifying association.

**Meaning:**

Based on this secondary analysis of a multicenter randomized trial, patients’ inflammatory status at admission was associated with their response to nutritional support and may be considered when individualizing the nutritional management of medical inpatients.

## Teaser

This secondary analysis of a randomized clinical trial examines whether patients’ baseline inflammatory status is associated with the effect of nutritional support on 30-day mortality among hospitalized adults.

## Introduction

Disease-related malnutrition is a frequent condition among hospitalized medical inpatients, with a prevalence of 20% to 50%.^1,2,3^ The 2019 Effect of Early Nutritional Support on Frailty, Functional Outcomes, and Recovery of Malnourished Medical Inpatients Trial* (*EFFORT)^4^ demonstrated that starting individualized nutritional support early reduces complications and mortality among medical inpatients at risk for malnutrition. Interestingly, there was little evidence in this trial for subgroup effects regarding nutritional status and type of medical disease. Nevertheless, independent of medical disease, patients’ inflammatory status could influence their response to nutritional support for several reasons.

Inflammation has several metabolic effects, including an increase in insulin resistance and reduction of appetite, leading to an inhibition of nutrition entering cells.^5,6^ In fact, independent of underlying disease, inflammation is thought to be a key driver for disease-related anorexia, reduced food intake, and muscle catabolism. This may also partly explain the inferior patient outcomes associated with inflammation, which include longer hospital stays and increased mortality.^7,8^ The relevance of inflammation in the pathogenesis of malnutrition is also reflected in its classification by the European Society of Clinical Nutrition and Metabolism (ESPEN). They recommend dividing malnutrition into disease-related malnutrition with and without inflammation.^9^ *Disease-related malnutrition with inflammation* is defined as underlying diseases causing inflammation with a consecutive lack of food intake or as uptake with a negative nutrient balance.^10^ Although several mainly preclinical studies have evaluated the relevance of inflammation on malnutrition, there is a lack of clinical data investigating whether the inflammatory status of a patient influences treatment response to nutritional support.

To close this gap, we conducted a secondary analysis of a prospective randomized clinical trial that included consecutive patients with malnutrition at the time of hospital admission. We investigated whether the inflammatory status of patients, as mirrored by their levels of C-reactive protein (CRP) at admission, was associated with treatment response within the trial and whether nutritional support was associated with CRP kinetics over time. Knowledge of such factors could improve our physiopathological understanding of the role nutrition plays during acute illness and may enable a more individualized nutritional approach to patients.

## Methods

### Study Design and Setting

This is a secondary analysis of EFFORT, a pragmatic, investigator-initiated, open-label, multicenter trial that was undertaken in 8 Swiss hospitals from April 2014 to February 2018. Between June and July 2019, we performed this secondary analysis. Reporting of the results follows the Consolidated Standards of Reporting Trials (CONSORT) reporting guideline for randomized clinical trials.^11^ The Ethics Committee of Northwestern Switzerland approved the study protocol, and all patients or their authorized representatives provided written informed consent. The main aim was to assess the effects of early nutritional support on patient outcomes in the medical inpatient setting. Rationale for the trial, design details, and eligibility features^12^ as well as the main results^4^ have been published previously. The trial protocol is available in Supplement 1.

### Patient Population and Management

In EFFORT, consecutive patients at nutritional risk (ie, Nutritional Risk Screening [NRS] 2002 total score ≥3 points^13^) with an expected hospital stay of at least 5 days were enrolled if they were willing to provide informed consent. Patients were excluded if they were initially admitted to intensive care units or surgical units; were unable to ingest oral nutrition; were already receiving nutritional support before admission; had a terminal condition (ie, end-of-life situation); were hospitalized because of anorexia nervosa, acute pancreatitis, acute liver failure, cystic fibrosis, or stem cell transplantation; had undergone gastric bypass surgery; had contraindications for nutritional support; or were previously included in the trial. While EFFORT included a total of 2028 patients, this secondary analysis included 1950 patients (96.2%) whose CRP levels were measured at time of admission as part of the clinical routine.

Upon admission, medical diagnosis according to *International Statistical Classification of Diseases and Related Health Problems, Tenth Revision *(*ICD*-*10*) codes, sociodemographic and anthropometric data, baseline muscle strength, and functional status (using the Barthel scale) were assessed in all patients based on the trial protocol. Following discharge, masked study nurses contacted patients after 30 and 180 days for a structured telephone interview. Prespecified health-related outcomes were systematically assessed at these points.

### Patient Groups and End Points

We allocated patients to 3 groups according to their inflammatory status at time of admission. Low inflammation was defined as CRP levels less than 10 mg/L, moderate as 10 mg/L to 100 mg/L, and high as greater than 100 mg/L (to convert CRP to nanomoles per liter, multiply by 9.524). These cutoffs were predefined based on a clinical rationale and prior experience with CRP levels among patients with various degrees of inflammation.^14^

Our main aim was to investigate whether a patient’s inflammatory status was associated with the effect of nutritional support on important outcomes. We compared different end points among patients receiving protocol-guided personalized nutritional support (ie, the intervention group) with those receiving standard hospital food (ie, the control group) within the predefined subgroups.

The primary end point was all-cause mortality after 30 days. Secondary end points were 180-day mortality, major complications, decline in functional status according to the Barthel scale at 30 and 180 days, and length of hospital stay. Adverse outcomes were defined as all-cause mortality, admission to intensive care units, and nonelective hospital readmission. The Barthel scale measures performance in activities of daily living and comprises 2 groups of items, 1 related to self-care (ie, feeding, grooming, bathing, dressing, bowel and bladder care, and toilet use) and the other to mobility (ie, ambulation, transfers, and stair climbing). We used the German version, which has scores ranging from 100 to 0, with lower scores indicating more severe disability. We defined decline as a reduction of 10% or more on the Barthel scale from time of admission.

### Statistical Analysis

Continuous variables were expressed as medians and interquartile ranges (IQRs), and frequencies were expressed as percentages and counts. We calculated logistic regression analysis and report odds ratios (ORs) and 95% CIs. We adjusted all analyses for predefined factors, including sex, age, baseline nutritional risk (ie, NRS 2002 score), study center, Barthel scale at baseline, main diagnosis, cardiovascular disease, renal disease, and cancer. We studied the effect of nutritional support overall and in subgroups by comparing outcomes among patients receiving nutritional support with control patients not receiving support. We included interaction terms in the statistical models to investigate whether there was evidence for effect modification due to baseline inflammatory status of patients (ie, low, moderate, or high inflammation). As a sensitivity analysis, we also included CRP as a continuous marker in the model. Finally, we performed a subgroup analysis limiting data to patients with a main diagnosis of infection to understand whether inflammation or infection was the main driver of results.

All statistical analyses were performed using Stata version 15.1 (StataCorp). *P* < .05 was considered statistically significant, and all tests were 2-tailed.

## Results

### Patient Population

**Figure 1.**
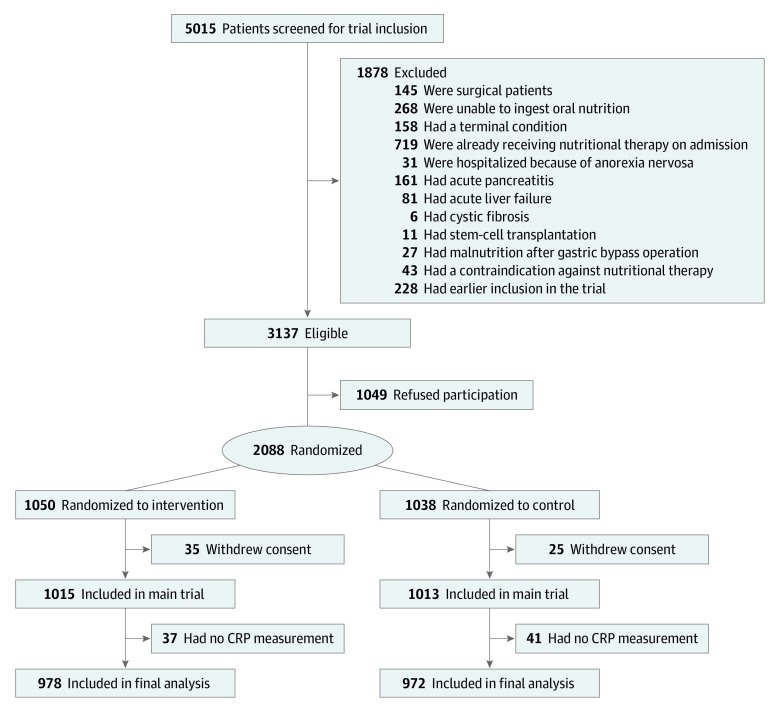
Flow of Patients Through the Trial CRP indicates C-reactive protein.

From an initial population of 2028 EFFORT trial patients (Figure 1), we had available CRP levels for 1950 patients (96.2%), of whom 533 (27.3%) had low levels of inflammation (CRP levels <10 mg/L), 894 (45.9%) had moderate levels of inflammation (CRP levels 10-100 mg/L), and 523 (26.8%) had high levels of inflammation (CRP levels >100 mg/L). Baseline characteristics for the overall population and those stratified according to inflammation status are shown in Table 1. The median (IQR) age of the population was 75 (65-83) years, and 1025 (52.6%) were men. All patients were at nutritional risk, with 598 (30.7%), 751 (38.5%), 499 (25.6%), and 102 (5.2%) having NRS 2002 scores of 3, 4, 5 and at least 6 points, respectively. The most common main diagnoses were infectious disease (592 [30.4%]), cancer (360 [18.5%]), and cardiovascular disease (197 [10.1%]), with significant differences among inflammation groups. For example, more patients with infectious diseases were in the high inflammation group than in the moderate or low inflammation groups (314 [60.0%] vs 235 [26.3%] vs 43 [8.1%]; *P* < .001), and more patients with cardiovascular disease were in the low inflammation group than in the moderate or high inflammation groups (72 [13.5%] vs 114 [12.8%] vs 11 [2.1%]; *P* < .001). The eTable in Supplement 2 also shows patient baseline and mean nutritional intake data during the hospital stay according to randomization group and stratified according to CRP group. Overall, baseline data were well balanced according to randomization groups within CRP groups. There were significantly higher mean calorie and protein intakes among patients in the intervention group compared with patients in the control group, regardless of CRP level (eg, high inflammation group: mean [SD] protein intake, 54.6 [23.5] g/d vs 44.7 [19.5] g/d; *P* < .001; mean [SD] calorie intake, 1432 [606] kcal/d vs 1138 [449] kcal/d; *P* < .001).

**Table 1.** Baseline Characteristics Overall and Stratified by Inflammation Level

Characteristic	No. (%)				*P* Value
	Overall (N = 1950)	CRP Levels <10 mg/L (n = 533)	CRP Levels 10-100 mg/L (n = 894)	CRP Levels >100 mg/L (n = 523)	
Age, median (IQR), y	75 (65-83)	74 (62-83)	76 (67-83)	74 (66-81)	.03
Men	1025 (52.6)	250 (46.9)	490 (54.8)	285 (54.5)	.009
BMI					
Median (IQR)	24.0 (21.0-28.0)	23.0 (20.0-27.0)	24.0 (21.0-28.0)	25.0 (21.5-28.0)	<.001
<18.5	173 (8.9)	72 (13.5)	67 (7.5)	34 (6.5)	<.001
18.5-25	1017 (52.3)	288 (54.0)	473 (53.1)	256 (49.2)	
>25	754 (38.8)	173 (32.5)	351 (39.4)	230 (44.2)	
NRS 2002 score					
3	598 (30.7)	198 (37.1)	294 (32.9)	106 (20.3)	<.001
4	751 (38.5)	211 (39.6)	328 (36.7)	212 (40.5)	
5	499 (25.6)	112 (21.0)	232 (26.0)	155 (29.6)	
≥6	102 (5.2)	12 (2.3)	40 (4.5)	50 (9.6)	
Main diagnosis					
Cardiovascular disease	197 (10.1)	72 (13.5)	114 (12.8)	11 (2.1)	<.001
Infectious disease	592 (30.4)	43 (8.1)	235 (26.3)	314 (60.0)	
Metabolic disorder	60 (3.1)	35 (6.6)	24 (2.7)	1 (0.2)	
Gastrointestinal disease	156 (8.0)	52 (9.8)	86 (9.6)	18 (3.4)	
Renal disease	66 (3.4)	18 (3.4)	38 (4.3)	10 (1.9)	
Cancer	360 (18.5)	89 (16.7)	174 (19.5)	97 (18.5)	
Pulmonary disease	117 (6.0)	31 (5.8)	62 (6.9)	24 (4.6)	
Neurological disorder	91 (4.7)	64 (12.0)	22 (2.5)	5 (1.0)	
Frailty	188 (9.6)	84 (15.8)	80 (8.9)	24 (4.6)	
Other	123 (6.3)	45 (8.4)	59 (6.6)	19 (3.6)	
Comorbidities					
Coronary heart disease	539 (27.6)	157 (29.5)	254 (28.4)	128 (24.5)	.15
Congestive heart failure	341 (17.5)	86 (16.1)	180 (20.1)	75 (14.3)	.01
Hypertension	1062 (54.5)	287 (53.8)	488 (54.6)	287 (54.9)	.94
Cerebrovascular disease	158 (8.1)	49 (9.2)	69 (7.7)	40 (7.6)	.56
Peripheral arterial disease	175 (9.0)	50 (9.4)	87 (9.7)	38 (7.3)	.27
Chronic kidney disease	618 (31.7)	147 (27.6)	308 (34.5)	163 (31.2)	.03
Diabetes	407 (20.9)	106 (19.9)	194 (21.7)	107 (20.5)	.69
COPD	291 (14.9)	75 (14.1)	143 (16.0)	73 (14.0)	.47
Dementia	72 (3.7)	24 (4.5)	31 (3.5)	17 (3.3)	.50
Malignant disease	647 (33.2)	143 (26.8)	309 (34.6)	195 (37.3)	<.001
Clinical findings					
Barthel scale					
Median (IQR)	90 (70-100)	90 (75-100)	90 (70-100)	85 (70-95)	<.001
<90 Points	1143 (58.6)	268 (50.3)	542 (60.6)	333 (63.7)	<.001
Admission CRP level					
Median (IQR)	34.0 (8.0-110.0)	4.0 (3.0-5.3)	35.0 (18.8-62.6)	172.0 (133.0-230.0)	<.001
Mean (SD)	71.8 (85.6)	4.3 (2.3)	41.9 (26.6)	191.5 (75.0)	<.001

Abbreviations: BMI, body mass index (calculated as weight in kilograms divided by height in meters squared); COPD, chronic obstructive pulmonary disease; CRP, C-reactive protein; IQR, interquartile range; NRS, Nutritional Risk Screening.

SI conversion factor: To convert CRP to nanomoles per liter, multiply by 9.524.

### Effect of Nutritional Support on 30-Day Mortality According to Inflammation Groups

**Figure 2.**
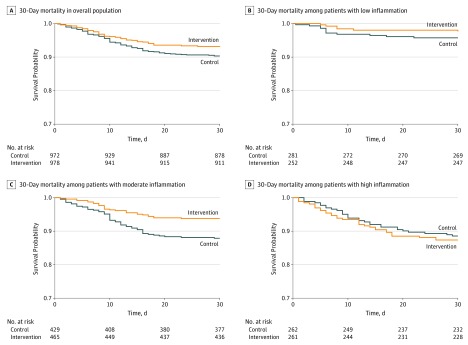
Kaplan-Meier Estimate for Time to Death Within 30 Days According to Inflammatory Status

Overall, there was a significant risk reduction for 30-day mortality among patients receiving nutritional support, with 7.0% (67 of 978) fulfilling the primary end point in the intervention group compared with 9.7% (94 of 972) in the control group (Figure 2). This effect was also confirmed by logistic regression analysis adjusted for sex, age, baseline nutritional risk (ie, NRS 2002 score), study center, Barthel scale at baseline, main diagnosis, cardiovascular disease, renal disease and cancer, with an adjusted OR of 0.61 (95% CI, 0.43-0.86; *P* = .005) (Table 2). To further understand whether baseline inflammation influenced the effect of nutritional support, we investigated mortality effects within subgroups based on inflammation and calculated interaction statistics. The effects of nutritional support remained robust among patients with low inflammation (adjusted OR, 0.34; 95% CI, 0.10-1.09; *P* = .02) and moderate inflammation (adjusted OR, 0.41; 95% CI, 0.24-0.68; *P* = .001) (Table 2). However, among patients with high inflammatory status, there was no significant benefit of nutritional support (adjusted OR, 1.32; 95% CI, 0.70-2.50; *P* = .39), with evidence for interaction (*P* for interaction = .005). Figure 2 shows a time-to-event analysis regarding the primary end point overall and stratified according to inflammation groups.

**Table 2.** Primary and Secondary Outcomes

End Point	Overall		CRP Level <10 mg/L			CRP Level 10-100 mg/L			CRP Level >100 mg/L		
	OR (95% CI)^a^	*P* Value	OR (95% CI)^a^	*P* Value	*P* Value for Interaction	OR (95% CI)^a^	*P* Value	*P* Value for Interaction	OR (95% CI)^a^	*P* Value	*P* Value for Interaction
30-d Mortality	0.61 (0.43 to 0.86)	.005	0.34 (0.10 to 1.09)	.02	.28	0.41 (0.24 to 0.68)	.001	.046	1.32 (0.70 to 2.50)	.39	.005
180-d Mortality	0.79 (0.63 to 1.01)	.06	0.72 (0.40 to 1.31)	.28	.29	0.78 (0.55 to 1.07)	.12	.86	0.95 (0.58 to 1.55)	.82	.57
Adverse outcome within 30 d	0.74 (0.59 to 0.91)	.006	0.79 (0.32 to 1.95)	.61	.76	0.66 (0.48 to 0.89)	.008	.52	0.82 (0.54 to 1.26)	.38	.93
ICU admission within 30 d	0.86 (0.47 to 1.58)	.63	0.67 (0.18 to 2.54)	.56	.56	1.09 (0.44 to 2.70)	.84	.28	0.68 (0.16 to 2.88)	.60	.51
Rehospitalization within 30 d	0.93 (0.68 to 1.29)	.67	1.16 (0.58 to 2.32)	.68	.34	0.80 (0.51 to 1.26)	.35	.55	0.90 (0.46 to 1.80)	.77	.74
Major complication within 30 d	0.92 (0.65 to 1.31)	.63	0.72 (0.29 to 1.78)	.46	.46	0.85 (0.53 to 1.36)	.50	.87	1.19 (0.59 to 2.38)	.63	.43
Barthel Scale decline, 30 d	0.65 (0.49 to 0.87)	.004	0.76 (0.36 to 1.61)	.48	.69	0.52 (0.35 to 0.78)	.002	.41	0.89 (0.50 to 1.56)	.67	.22
Barthel Scale decline, 180 d	1.00 (0.81 to 1.24)	.98	1.55 (0.99 to 2.41)	.05	.21	0.91 (0.67 to 1.24)	.56	.51	0.83 (0.52 to 1.32)	.44	.49
Length of hospital stay, d	−0.33 (−0.89 to 0.23)	.25	−0.40 (−1.37 to 0.57)	.42	.56	−0.25 (−1.09 to 0.59)	.55	.55	−0.46 (−1.71 to 0.79)	.47	.85

Abbreviations: CRP, C-reactive protein; ICU, intensive care unit; OR, odds ratio.

SI conversion factor: To convert CRP to nanomoles per liter, multiply by 9.524.

^a^ Adjusted for randomization, sex, nutritional risk screening score, study center, Barthel scale, main diagnosis, cardiovascular disease, renal disease, and cancer.

In a sensitivity analysis, we also found that when CRP was included in the model as a continuous variable, there was evidence for effect modification for CRP on the association of nutritional support and mortality (*P* for interaction = .005). We also performed a subgroup analysis limited to 592 patients (30.4%) with a systemic infection as their main admission diagnosis. Within this subgroup, the strength of the association of nutritional support with 30-day mortality again differed among CRP groups with adjusted ORs of 0.78 (95% CI, 0.05-13.40; *P* = .88) and 0.51 (95% CI, 0.17-1.53; *P* = .23) for the low and moderate CRP groups, respectively, and an adjusted OR of 1.24 (95% CI, 0.51-3.00; *P* = .64) for the high CRP group.

### Effect of Nutritional Support on Secondary Outcomes According to Inflammation Groups

We also conducted several analyses to investigate the association of nutritional support with different secondary short-term outcomes measured at 30 days and long-term outcomes measured at 180 days (Table 2). For 180-day mortality and major complications, patients with high inflammation on admission tended to benefit less from nutritional support (180-d mortality: adjusted OR, 0.95; 95% CI, 0.58-1.55; *P* = .82; major complications: adjusted OR, 1.19; 95% CI, 0.59-2.38; *P* = .63), but these results were not significant in the interaction analysis. For other secondary end points, results remained robust with no evidence of interaction due to baseline inflammation status.

### Association of Nutritional Support With CRP Kinetics

**Figure 3.**
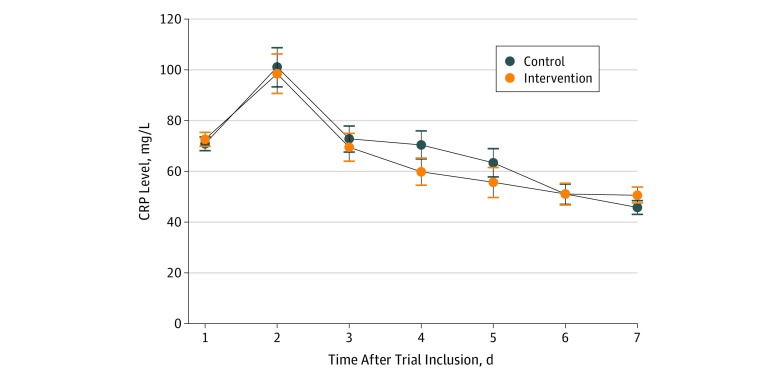
C-Reactive Protein (CRP) Kinetics Within the First 7 Days of Inpatient Treatment According to Randomization Group To convert CRP to nanomoles per liter, multiply by 9.524.

Finally, we investigated whether nutritional support was associated with CRP kinetics during the first 7 days of inpatient treatment. As shown in Figure 3, CRP levels increased from baseline to day 2 and thereafter decreased stepwise with no difference between patients in the intervention group and the control group at any day. The mean (SD) CRP levels for the control and intervention groups on day 1 were 70.93 (2.72) mg/L and 72.61 (2.77) mg/L, respectively. On day 2, these increased to 101.08 (7.72) mg/L and 98.48 (7.80) mg/L, respectively, before decreasing to 45.76 (2.74) mg/L and 50.66 (3.09) mg/L, respectively, on day 7.

## Discussion

The key findings of this secondary analysis investigating the effect of nutritional support among hospitalized patients according to their baseline inflammatory status are 2-fold. First, we found that patients with high baseline inflammation (ie, CRP levels >100 mg/L) were not associated with a benefit from nutritional support with regard to 30-day mortality (ie, the primary end point of this analysis), with a significant result in interaction analysis. Patients with low and moderate inflammation were associated with a significant reduction in 30-day mortality, similar to the overall population. These results remained similar in a subgroup analysis limited to patients with a systemic infection as main admission diagnosis, suggesting that inflammation rather than infection was the main driver of results. Second, we did not find that nutritional support was associated with inflammation as mirrored by a similar kinetic profile of CRP levels over the first 7 days of inpatient treatment among individuals with and without nutritional support.

Recently, there have been several studies showing that nutritional support has a positive effect on clinical outcomes among patients with malnourishment, particularly among medical patients with multiple illnesses and comorbidities.^4,15,16^ Nevertheless, because trials in some populations have reported negative results, it has been hypothesized that not all patients would have the same response to nutrition, emphasizing the concept of personalized nutrition.^17,18,19^ Inflammation could be a key factor, which could explain these differences.^20^ In fact, there is a strong biological explanation for why inflammation is associated with the effect of nutritional support on patient outcomes. Previous studies have found that inflammation because of acute or chronic disease causes metabolic changes by influencing different pathways.^21,22^ Inflammation has effects on appetite and food intake, gastrointestinal functioning of the stomach and gut, and, on a cellular level, on insulin resistance, among others.^23^ Among other mechanisms, these effects are mediated by circulating cytokines released as part of the systemic inflammatory response.^24^ Their stress-response release during illness plays an integral role in the systemic inflammatory response, and several studies have found cytokines to be associated with disease-related anorexia, weight loss, decline in cognitive function, frailty, and anemia. For example, interleukin-6 and tumor necrosis factor-α interact with brain circuitries that control food intake, delayed gastric emptying, and muscle catabolism.^25^ Interestingly, the cytokine-induced downregulation of food intake during acute illness may also have a beneficial biological role, given that high intake of nutrition during severest illness (ie, overfeeding) has been shown to reduce autophagy, a mechanism important for cell detoxication during illness.^26,27,28^ These observations have also been confirmed in several clinical trials that report no benefit from full-replacement feeding among patients who are critically ill.^29,30^ The findings of our study, which looked at a lower-risk patient population in medical wards, are in line with these observations. Overall, they demonstrate that patients do benefit from nutritional support, but those with initially very high levels of CRP and thus marked inflammation may not respond. Of note, our subgroup analysis limited to patients with systemic infection also suggests that it is inflammation and not infection that is driving these results. Individualized nutritional support in these cases even tends to have a harmful effect on 30-day mortality. This finding would also be in line with data observed in patients who are critically ill, who typically have a very strong systemic inflammatory response. Thus, our findings support the concept of individualized nutritional support based on a patient’s initial presentation and markers of inflammation, possibly with lower targets for those patients with higher baseline levels of inflammation. However, this hypothesis needs confirmation in prospective trials.

We also investigated whether the association of inflammation and nutrition was bidirectional, ie, whether nutritional support would also be associated with inflammation mirrored by the kinetics of CRP levels over time. However, there was no difference in inflammation during the first 7 days of inpatient treatment for those with and without nutritional support. Thus, there is no evidence from our analysis that the modulation of inflammation through nutritional support in acute situations would be responsible for the positive effects seen on outcomes in previous trials. It is still possible that nutrition has beneficial effects in chronic situations, eg, by modulation of low-grade inflammation. Future trials should look at this particular question to better understand the physiopathology regarding the effects of nutrition on outcome.

### Strengths and Limitations

To our knowledge, this is the first large-scale analysis to investigate whether inflammation is associated with response to nutritional support based on a secondary analysis of a randomized clinical trial. However, we are aware of several limitations. First, we only measured CRP levels and did not look at other cytokines, which could have delivered more detailed information. Second, the sample size may have been too small to find significant interactions in some of the outcomes investigated. Third, we did not adjust our analysis for all possible confounders; therefore, there could still be a residual confounding of our analysis. Fourth, because it is a secondary analysis, our results are hypothesis generating rather than definite and require validation in an independent sample.

## Conclusions

Based on this secondary analysis of a multicenter randomized clinical trial, patients’ inflammatory status at admission was associated with their response to nutritional support. These findings may help to better individualize nutritional support based on patients’ initial presentation and markers of inflammation.

## Notes

Supplement 1. **Trial Protocol**

Supplement 2. **eTable.** Baseline Characteristics According to Randomization Arm Overall and Stratified by CRP Status

Supplement 3. **Data Sharing Statement**
