# Supplementary material for: Association of Baseline Inflammation With Effectiveness of Nutritional Support Among Patients With Disease-Related Malnutrition: A Secondary Analysis of a Randomized Clinical Trial
Source: JAMA Netw Open. 2020 Mar 10;3(3):e200663. doi: 10.1001/jamanetworkopen.2020.0663 (PMC7064875; doi:10.1001/jamanetworkopen.2020.0663)

Title page

Full Title: Effect of Early Nutritional Therapy on Frailty, Functional Outcomes and Recovery of Undernourished Medical Inpatients Trial: The EFFORT Project

Short Title: Effect of Early Nutritional Therapy on Frailty, Functional Outcomes and Recovery of Medical Inpatients

Principal Investigator and Sponsor:
Prof. Dr. med. Philipp Schuetz, M.D., MPH
Department of Endocrinology, Diabetes and Clinical Nutrition
University Department of Internal Medicine,
Kantonsspital Aarau, Tellstrasse, CH-5001 Aarau, Switzerland
Email: Philipp.Schuetz@unibas.ch

Study type: Randomised controlled trial

Study category: A (Risk category according to LHR)

Funding: This study is funded by the Swiss National Foundation (SNF, PP00P3_150531/1)

Document Status: first review 01/2014 by EKNZ (Ref. Nr. 2014_001)

Clinical Trial Number: *not yet registered*

Protocol version and date: 3.3 (13/01/2020)

Confidentiality statement
The information contained in this document is confidential. The information may not - in full or in part - be transmitted, reproduced, published, or disclosed to others than the applicable Competent Ethics Committee(s) and Regulatory Authority(ies) without prior written authorisation from the sponsor except to the extent necessary to obtain informed consent from those who will participate in the study.

Co-Investigators

For the randomized, controlled trial

- University Department of Internal Medicine, Nursing Department & Ernährungsberatung, Kantonsspital Aarau (Dr.M.Bally, Prof.B.Mueller, M.Deiss, R.Fehr, C. Siebenthal-Wehrli, B.Reutlinger, A.Kutz, S.Felder)
- Department of Internal Medicine, Inselspital Bern, Switzerland (PD Dr. Z.Stanga, PD Dr. M.Bodmer, Dr. M.Leuenberger)
- Department of Internal Medicine, Spital Zofingen, Switzerland (Dr.T.Ehmann, Dr. B.Flückiger, S.Becker)
- Department of Internal Medicine, Kantonsspital Luzern, Switzerland (PD Dr.C.Henzen, M.Nigg, K.Bucher)
- Department of Endocrinology, Diabetes, and Metabolism, University Hospital Basel (Prof. M. Christ-Crain, Prof. M.Donath, Prof U.Keller)
- Medical University Clinic, Departement of Gastroenterology, Hepatology, and Nutrition, Kantonsspital Baselland, Liestal (Prof. R.Meier)
- Internal Medicine und Ernährungsberatung, Spital Winterthur (A. Uster, M. Ruehlin, Dr. R. Imoberdorf)
- Department of Internal Medicine, Spital Muri (Dr. A.Spillmann, PD Dr. V.Kaplan, T.Keller)
- Gesellschaft für Klinische Ernährung Schweiz (GESKES) (Prof. P.Ballmer)
- Clinical Trial Unit (CTU), University of Basel (Dr. T.Fabbro, Dr. S. von Felten)
- Department of Human Nutrition, Faculty of Life Sciences, University of Copenhagen, Clinical Nutrition Unit, Rigshospitalet University Hospital, Copenhagen, Denmark (Prof. J.Kondrup)
- Department of Internal Medicine and Service d'Endocrinologie, Diabétologie et Métabolisme, CHUV, Lausanne (Prof F. Pralong, Dr. P. Coti)
- University Hospitals Leuven (UZ Leuven) (Prof. G. Van den Berghe, Dr. M. Casaer)

For the ancillary Projects

- Department of Psychology, University of Berne (Prof. P. Perrig-Chiello)
- Institute of Nursing Science, University of Basel (Prof. S. De Geest)
- Department of Endocrinology, University of Fribourg (Prof. A. Lauber)

Signature Page

Study number: EKNZ 2014_001

Study title: Effect of Early Nutritional Therapy on Frailty, Functional Outcomes and Recovery of Undernourished Medical Inpatients Trial: The EFFORT Project

The Sponsor-Investigator and trial statistician have approved the protocol version [3.3 (27.3.2014)], and confirm hereby to conduct the study according to the protocol, current version of the World Medical Association Declaration of Helsinki, ICH-GCP guidelines or ISO 14155 norm if applicable and the local legally applicable requirements.

Sponsor and principal investigator:

**Contact Person**	**Prof. Philipp Schütz**		
**Function**	**Leitender Arzt**		
**Institution**	**Medizinische Universitätsklinik der Universität Basel**<br><br>**Kantonsspital Aarau,**		
**Address**	**Tellstrasse CH -5001 Aarau, Switzerland**		
**Date**	**1/4/2014**	**Signature**	Philipp Schütz

Investigator's Agreement

Local Principal Investigator at study site*:

I have read and understood this trial protocol and agree to conduct the trial as set out in this study protocol, the current version of the World Medical Association Declaration of Helsinki, ICH-GCP guidelines or ISO 14155 norm and the local legally applicable requirements.

Site ______________________________

Principal investigators ______________________________

______________________ ______________________

Place/Date Signature

**Note:* In multicentric studies, this page must be individually signed by all participating Local Principal Investigators.

Table of contents

SYNOPSIS - 6 -
ABBREVIATIONS - 8 -
1. STUDY ADMINISTRATION AND LEGAL ASPECTS - 9 -
1.1. ROLE OF FUNDING - 9 -
1.2. STATISTICAL ANALYSIS - 9 -
1.3. STUDY REGISTRATION - 9 -
1.4. CATEGORISATION OF THE STUDY - 9 -
1.5. COMPETENT ETHICS COMMITTEE AND ETHICAL CONDUCT OF THE STUDY - 9 -
1.6. DECLARATION OF INTEREST - 9 -
1.7. PATIENT INFORMATION AND INFORMED CONSENT - 10 -
1.8. EARLY STUDY TERMINATION - 10 -
2. BACKGROUND AND RATIONAL - 11 -
2.1. TABLE 1 - STUDY OVERVIEW - 12 -
2.2. RISK BENEFIT - 13 -
2.3. CHOICE OF STUDY POPULATION - 13 -
3. OBJECTIVES AND PURPOSE - 14 -
3.1. Overall hypothesis - 14 -
3.2. Objective - 14 -
3.3. Setting - 14 -
4. DESIGN OF THE TRIAL - 14 -
4.1. Study design - 14 -
4.2. Primary, secondary and safety endpoints - 15 -
4.4. Initial patient assessment - 16 -
5. SELECTION OF TRIAL SUBJECTS - 17 -
5.1. Recruitment - 17 -
5.2. Inclusion criteria - 17 -
5.3. Exclusion criteria - 17 -
6. ASSESSMENT OF EFFECTIVENESS - 20 -
6.1. Intervention - 20 -
6.2. Blinding - 20 -
7. ASSESSMENT OF SAFETY - 21 -
8. STATISTICS - 21 -
8.1. Analysis population - 21 -
8.2. Statistical approach - 21 -
8.3. Sample Size considerations - 22 -
9. TRIAL-SPECIFIC PREVENTIVE MEASURES AND DUTIES - 23 -
9.1. Trial monitoring - 23 -
9.2. Adverse outcome - 24 -
10. DUTIES ON THE PART OF THE INVESTIGATOR - 26 -
11. ETHICAL CONSIDERATIONS - 27 -
12. QUALITY CONTROL AND ASSURANCE: DESCRIPTION OF MEASURES - 28 -
12.1. QUALITY ASSURANCE AND DATA/RECORD KEEPING - 28 -
13. ANCILLARY PROJECTS - 29 -
13.1. Physio-pathological mechanisms of action of nutritional therapy - 29 -
13.2. Cost-effectiveness analysis of nutritional therapy - 29 -
13.3. Impact of nutritional support on glucose metabolism and insulin regimens - 31 -
13.4. Biosampling for later detection of genetic modulators - 31 -
13.5. Importance of nurse-sensitive factors - 32 -
14. SCHEDULE AND MILESTONES - 33 -
15. POTENTIAL LIMITATIONS - 33 -
16. IMPORTANCE AND IMPACT OF THE PROPOSED PROJECT - 34 -
17. REFERENCES - 36 -

Synopsis

**Sponsor / Sponsor-Investigator**	*Prof. Dr. med. Philipp Schuetz, M.D., MPH; Department of Endocrinology, Diabetes and Clinical Nutrition; University Department of Internal Medicine, Kantonsspital Aarau, Tellstrasse, CH-5001 Aarau, Switzerland*
**Study Title:**	*Full Title: Effect of Early Nutritional Therapy on Frailty, Functional Outcomes and Recovery of Undernourished Medical Inpatients Trial: The EFFORT Project*
**Short Title**	*The EFFORT trial*
**Protocol Version**	*EKNZ 2014_001 (version 3.3, 27.3.2014)*
**Trial registration:**	*This trial will be registered at www.ClinicalTrials.gov*
**Study category and Rationale**	*Category A (nutritional products used are authorised in Switzerland and the application is done according to the specialised information*
**Clinical Phase:**	*Confirmation study regarding effectiveness and safety of nutritional therapy in medical inpatients*
**Background and Rationale:**	*Illness is associated with appetite loss leading to relevant nutritional deficits, particularly in elderly, frail medical inpatients. Undernutrition is associated with morbidity, long hospital stays and costs. Despite these associations, benefit of providing early nutritional therapy to unselected medical inpatients at risk for undernutrition remains largely unproven due to lack of large-scale, high-quality randomized controlled trials (RCTs). Therefore, a large conclusive trial is urgently needed to answer the questions (a) whether or not appetite loss during acute illness is indeed a protective physiological response that should be tolerated; (b) which patients do or do not benefit from nutritional therapy; (c) how and why nutrition affects the course of disease from a mechanistic physio-pathological standpoint.*
**Objective(s):**	*To test the hypothesis that in medical inpatients at risk for undernutrition defined by the nutritional risk score (NRS 2002), early tailored nutritional therapy to reach nutritional targets based on individualized nutritional counseling is a cost-effective strategy to prevent mortality, morbidity and functional decline.*
**Outcome(s):**	*The primary composite endpoint is combined adverse outcome within 30 days defined as (a) all-cause mortality, (b) admission to the intensive care unit from the medical ward, (c) major complications, (d) unplanned hospital readmissions and (d) decline in functional outcome from admission to day 30 assessed by Barthel`s index (-10%). Secondary endpoints include (a) each single component of the primary endpoint (b) short-term nutritional and functional outcomes from inclusion to day 10 or hospital discharge; (c) hospital outcomes; (d) 30-day and 180-day outcomes (e) Other safety endpoints including adverse gastrointestinal effects associated with nutritional therapy assessed daily until hospital discharge.*
**Study design:**	*Randomized controlled trial*
**Inclusion / Exclusion criteria:**	*Unselected adult medical inpatients at risk of undernutrition [NRS≥3 points] and an expected hospital stay of ≥5 days who are willing to provide informed consent will be included. We will exclude patients in critical care or post-operative state, unable to swallow, at long-term need for parenteral/enteral nutrition, in terminal condition, pregnant, with acute pancreatitis or acute liver failure, with anorexia nervosa, that were earlier included into the trial*

**Measurements and procedures:**	*Patients in the intervention group will receive individualized nutritional therapy to reach nutritional targets (caloric, protein, micronutrients, other) based on a predefined nutritional strategy. In control patients, according to patients` appetite, standard hospital nutrition will be served. Nutritional therapy may be started in control patients, if any sort of swallowing disorders develops or if patients need to be prepared for operation. All patients will be re-assessed daily during the hospital stay for nutritional intake and nutritional therapy may be escalated every 24-48 hours (food fortification, oral supplements, enteral, parenteral nutrition) if targets are not met (at least 75% of targets).*
**Study Product / Intervention:**	*Different nutritional products (oral nutritional supplements, enteral nutrition, parenteral nutrition) to reach nutritional targets*
**Control Intervention:**	*Usual care;*
**Number of Participants with Rationale:**	*The inclusion of 2000-3000 patients will provide 78-90% power to detect a reduction in the primary endpoint of 15% (from 40% to 34%)*
**Study Duration:**	*After a 3 month pilot (feasibility period), other centers will be stepwise included for a total duration of 3 years, or until the total sample size is included.*
**Study Schedule:**	*05/2014Year of First-Participant-In (planned)*<br>*05/2017 Year of Last-Participant-Out (planned)*
**Investigator(s):**	*This project is embedded in multiple research national and international collaborations with nutritional experts. The RCT will be conducted in multiple experienced Swiss centers and possibly international sites. The Clinical Trial Unit (CTU) of the University of Basel will provide logistical and statistical support.*
**Study Centre(s):**	*Single-centre or multi-centre. If multi-centre note number of projected centres to be involved. Or countries if multi-national study*
**Outlook and discussion**	*Important gaps in the literature and the controversy about nutritional therapy in critical illness call into question our current nutritional therapy approach in medical inpatients. Herein, integrating recognized international experts and generating novel data both from the current literature and state-of-the-art investigation involving thousands of patients, we aim to establish an evidence-based standard for nutritional strategy for acutely-ill medical inpatients. This strategy will be tested in the largest nutritional RCT yet outside critical care and will allow us to answer whether, why, how, and where nutritional therapy works. This “real-world” comparative effectiveness research project thus has the potential to provide conclusive evidence about nutritional therapy in unselected, acutely-ill hospitalized, often polymorbid and frail medical inpatients to facilitate informed decision-making by patients and healthcare professionals world-wide.*
**GCP Statement:**	*This study will be conducted in compliance with the protocol, the current version of the Declaration of Helsinki, the ICH-GCP or ISO EN 14155 (as far as applicable) as well as all national legal and regulatory requirements.*

ABBREVIATIONS

AE	Adverse Event
BMI	body mass index
BMR	Basal metabolic rate (BMR)
BIA	Body impedance
CRF	Case Report Form
CTU	Clinical trial unit
eCRF	Electronic Case Report Form
FAACT	Functional Assessment Anorexia-Cancer Therapy
GCP	Good Clinical Practice
GI	Gastro-intestinal
IIT	Investigator-initiated Trial
IL	Interleukin
ITT	Intention to treat
ONS	Oral nutrition supplement
PI	Principal Investigator
QoL	Quality-of-life
RCT	Randomised controlled trial
SOP	Standard Operating Procedure
TNF	Tumor nekrosis factor
LOS	length-of-stay

1. Study Administration and legal aspects

1.1. Role of funding

This trial is supported by the Swiss National Science Foundation (SNSF Professorship, PP00P3_150531 / 1). Additional funding will be requested from hospital Institutions (ie, Kantonsspital Aarau) and Industrial partners.

1.2. Statistical analysis

For the statistical analysis, the investigators will closely collaborate with the clinical trial unit of the University of Basel (Dr. Thomas Fabbro and Dr. Stephanie von Felten, CTU, University of Basel, Basel, Switzerland).

1.3. Study registration

The study will be registered at the ClinicalTrials.gov website.

1.4. Categorisation of the study

This trial correspond to the Category A because the different nutritional products used are authorised in Switzerland and the application is done according to the specialised information.

1.5. Competent Ethics Committee and ethical conduct of the study

The main ethical committee will be the EKNZ (Leitethikkomission, EKNZ 2014_001).

The responsible investigator at each site ensures that approval from an appropriately constituted Competent Ethics Committee is sought for the clinical study.

All changes in the research activity and all unanticipated problems involving risks to humans will be reported to the all ethical committees within 48 hours. Premature study end or interruption of the study is reported within 15 days. The regular end of the study is reported to the CEC within 90 days, the final study report shall be submitted within one year after study end.

The study will be carried out in accordance to the protocol and with principles enunciated in the current version of the Declaration of Helsinki, the guidelines of Good Clinical Practice (GCP) issued by ICH, in case of medical device: the European Directive on medical devices 93/42/EEC and the ISO Norm 14155 and ISO 14971, the Swiss Law and Swiss regulatory authority's requirements. The CEC and regulatory authorities will receive annual safety and interim reports and be informed about study stop/end in agreement with local requirements.

1.6. Declaration of interest

All coinvestigators will declare their conflict of interest as appropriate.

1.7. Patient information and informed consent

The investigators or other authorised individuals personal will explain to each participant the nature of the study, its purpose, the procedures involved, the expected duration, the potential risks and benefits and any discomfort it may entail. Each participant will be informed that the participation in the study is voluntary and that he/she may withdraw from the study at any time and that withdrawal of consent will not affect his/her subsequent medical assistance and treatment.

The participant must be informed that his/her medical records may be examined by authorised individuals other than their treating physician. All participants for the study will be provided a participant information sheet and a consent form describing the study and providing sufficient information for participant to make an informed decision about their participation in the study. The patient information sheet and the consent form will be submitted to the ethical review board to be reviewed and approved. The formal consent of a participant, using the approved consent form, must be obtained before the participant is submitted to any study procedure.

The participant should read and consider the statement before signing and dating the informed consent form, and should be given a copy of the signed document. The consent form must also be signed and dated by the investigator (or his designee) and it will be retained as part of the study records.

The investigator affirms and upholds the principle of the participant's right to privacy and that they shall comply with applicable privacy laws. Especially, anonymity of the participants shall be guaranteed when presenting the data at scientific meetings or publishing them in scientific journals.

Individual subject medical information obtained as a result of this study is considered confidential and disclosure to third parties is prohibited. Subject confidentiality will be further ensured by utilising subject identification code numbers to correspond to treatment data in the computer files.

For data verification purposes, authorised representatives of the Sponsor (-Investigator), a competent authority or an ethics committee may require direct access to parts of the medical records relevant to the study, including participants' medical history.

1.8. Early study termination

The Sponsor-Investigator may terminate the study prematurely according to certain circumstances, for example:

- ethical concerns,
- insufficient participant recruitment,
- when the safety of the participants is doubtful or at risk, respectively,
- alterations in accepted clinical practice that make the continuation of a clinical trial unwise,
- early evidence of benefit or harm of the experimental intervention

2. Background and rational

Acute and chronic illness is associated with loss of appetite and body weight, which increases the risk for undernutrition, particularly in the elderly and frail medical patient population[1]. This relationship between acute disease and eating behavior / nutritional status may well be bi-directional, with illness affecting nutritional status, but also dietary factors influencing the course of illness. For example, cytokines, such as interleukin (IL)-6 and tumor-necrosis-factor (TNF)-alpha, affect brain circuitries that control food intake, delayed gastric emptying and skeletal muscle catabolism[2, 3]. Whether loss of appetite associated with acute illness is indeed a protective physiological response or a therapeutic target needing early corrective nutritional therapy is debated. Such controversy over the benefits and optimal protocols of nutritional therapy can only be resolved with a large randomised-controlled trial (RCT) comparing early nutritional therapy with “appetite-guided” nutrition in this population.

Undernutrition is common in elderly, chronic and/or polymorbid inpatients and associated with detrimental metabolic consequences, such as catabolism and muscle wasting[1]. Undernutrition *per se* is associated with higher mortality and morbidity, increased risk for infection and an increased hospital length-of-stay (LOS)[4, 5]. Despite a shortfall of evidence that nutritional therapy actually alters clinical outcomes, these relationships have led to the current clinical approach of providing nutritional therapy early to medical inpatients at risk for undernutrition as an empiric strategy to combat undernutrition and associated adverse outcomes. However, apart from critical care, where large trials recently have been published[6-8], there is an important lack of high-quality data from large RCTs in acutely-ill medical inpatients to shed light on the optimal type, caloric amount and timing of such therapy.

Smaller trials have investigated the effects of nutritional strategies on selected patient outcomes, mainly changes in body weight and nutrition-specific quality of life (*Table 1*). Some trials suggested that clinical outcomes are improved by nutritional therapy in regard to risk of infections, complications, lengths of hospital stay, functional status (activities of daily living), quality of life and even mortality. However, most trials were highly heterogeneous in design, patient populations and type of interventions, lacked statistical power to demonstrate safety and together, produced inconclusive results. Unsurprisingly, two previous aggregate data meta-analyses confirm the important lack of high-quality evidence to endorse or refute nutritional support[9, 10]. Both meta-analyses, however, were based on aggregate data only and did not specifically examine the effect of early nutritional therapy in medical inpatients. Rather, one meta-analysis focused on such therapy in critical care/perioperative patients[9], while the other only considered general protein and energy supplementation in the elderly[10]. Moreover, having been published in 2007 and 2009, respectively, neither meta-analysis captures the most recent work. We are currently performing an up-to-date meta-analysis based on aggregate data and individual patient data (IPD), to provide a solid evidence base for the development of a “state-of-the-art” nutritional therapy strategy, which will be tested in the largest yet randomized controlled intervention trial outside critical care.

2.1. Table 1 - study overview of most recent RCTs evaluating nutritional therapy in medical inpatients

First author year	Patient type	N	Study intervention	Control treatment	Outcomes evaluated	Main effects of study Intervention	Limitations	Ref
**Somanchi, 2011**	Adult hospitalized medical patients	400	Individualized nutritional counseling	Standard nutritional care	LOS, diagnosis coding of malnutrition cases delays in nutritional support	Significant reduction in LOS with economic benefit	No patient level randomization	11
**Starke J 2011**	Malnourished general medical inpatients	132	Individualized ONS	Standard nutritional care	Caloric intake, weight vitamin levels, QoL complications, readmission, mortality (6m)	Higher caloric/protein intake, less weight loss increased QoL, fewer complications	Small sample time-consuming intervention	12
**Rüfenacht U, 2010**	Malnourished general medical inpatients	36	Intensive nutritional counseling with ONS	ONS only	Anthropometrics, energy and protein intake, QoL questionnaire	Higher caloric/protein intake, QoL improvement after hospitalization	Small sample, two intervention groups	13
**Norman K 2008**	Malnourished inpatients with GI disease	101	Dietary counseling with ONS over 3-month period	Dietary counseling without ONS	BMI, muscle strength readmission, QoL	Improved hand grip strength and peak flow fewer readmissions, better QoL	High dropout rate two intervention groups	14
**Gariballa SA, 2007**	Acutely ill inpatients >65y	225	400 ml ONS/d	Standard nutritional care plus placebo	Nutritional status depression (geriatric depression questionnaire), cognitive function	Significant improvement in depression score after 6 month, no difference in cognitive function	High lost to follow number	15
**Gariballa S 2006**	Malnourished inpatients >65y	445	400 ml ONS/d	400 ml placebo/d	Barthel's index readmissions, LOS mortality	Fewer readmissions otherwise no effect	Low adherence of intervention	16
**Johansen N, 2004**	Malnourished inpatients	212	Individualized nutritional therapy	Standard nutritional care	Caloric intake, LOS complications, mortality QoL	Higher caloric/protein intake, shorter LOS	Small sample	17
**Hickson M 2004**	Malnourished medical inpatients, >65 y old	592	Nutritional counseling by health care assistants	Standard nutritional care	Weight/BMI, Barthel`s index, infection, LOS, in-hospital mortality	Less antibiotic use otherwise no effect	Short training for health care assistants (15h)	18

BMI, body mass index; d, day(s); GI, gastro-intestinal; h, hours(s); LOS, length-of-stay; m, month(s); ONS, oral nutrition supplement; QoL, quality-of-life

2.2. Risk benefit

Despite the absence of high-quality RCT data, the current consensus clinical approach in unselected medical inpatients is to provide nutritional therapy to reach nutritional requirements including caloric and protein targets, as well as micro-nutritional requirements. Importantly, some recent data from critical care suggested even harmful effects of aggressive early feeding, as well as glutamine supplementation which may be explained by different reasons[6, 7, 19]. During the acute phase of illness, the body mobilizes substrates from muscle and fat tissue to match the increased resting energy expenditure[20]. Exogenous calories then no longer inhibit gluconeogenesis. Excessive nutrition during the acute phase of illness can thus induce occult overfeeding. Still, recent critical care research from Switzerland reported benefit from individually optimized energy supplementation when patients were well selected[8]. The contradictory findings from these trials may be partly explained by the differences in patient population, nutritional strategy and trial design. A key consideration which not only applies to critical care is therefore the fact that every ill patient is different, and nutritional strategies and goals need to be personalized and tailored to individual requirements[20].

Importantly, data from critical care cannot unconditionally be transferred to medical inpatients with a lower degree of disease severity. Still, the above-mentioned conflicting observations re-emphasize that nutritional therapy is a medical intervention with associated risks and costs, and call into question todays nutritional approach in medical inpatients. The current lack of strong guideline recommendations[21-34] for type, caloric amount and timing of nutritional therapy in acutely-ill medical inpatients outside critical care is mainly explained by the paucity of high-level evidence showing such therapy’s efficacy, safety and cost benefits, and the absence of knowledge regarding which patient population do or do not benefit. Hence, evaluation of efficacy, safety and possible cost benefits within a large, well-controlled conclusive RCT is warranted to assess the effects of early nutritional therapy on patient outcomes in the medical inpatient setting. The EFFORT trial will not only answer the question about overall benefit or harm, but using a physio-pathological mechanistic approach, it also will explore and provide conclusive answers about whether, why, how, and in which patient populations nutritional therapy does or does not works.

2.3. Choice of study population

Most current nutritional research has focused on selected medical diseases (e.g., pancreatitis). Consequently, these “clean” results may not be generalizable to “real-life” unselected medical inpatients with multiple comorbidities and illnesses. Comparative effectiveness research aims at improving quality, effectiveness, and efficiency of health care and at helping patients and healthcare professionals make informed decisions[35]. To achieve these goals, research must address the patient population that actually consumes the most health care, specifically polymorbid, frail, elderly patients with complex combinations of medical diagnoses. Although accounting for the majority of costs, this patient population is the least

studied[36] To correct this disparity, clinical trials should include large, representative populations, to enable examination of treatment effects within key subpopulations, and to allow robust head-to-head comparison of interventions[35].

EFFORT will close this important gap. The project focuses on a major issue in hospital care, namely, whether, how and why early nutritional therapy affects outcomes of unselected, elderly, frail acutely-ill medical inpatients. Evidence generated by this project will therefore easily be transferable to clinical practice and thus can be expected to exert a major direct impact on current patient management.

3. Objectives and purpose

3.1. Overall hypothesis

Our overall hypothesis states that in acutely-ill medical inpatients at risk for undernutrition, early nutritional therapy to reach nutritional targets is a cost-effective strategy to prevent mortality, morbidity and decline in functional outcomes. The alternative hypothesis is that early nutritional therapy has no beneficial effects, but increases the risk of complications, hospital LOS and overall treatment costs.

3.2. Objective

Our aim is to conduct a large investigator-initiated, non-commercial RCT to compare a “state-of-the-art” individualized nutritional therapy strategy to cover nutritional requirements based on pre-specified nutritional therapy guidelines (intervention group) versus a control group without nutritional therapy in a large representative medical inpatient population at risk for malnutrition.

3.3. Setting

Several secondary and tertiary Swiss hospitals have agreed to participate in this multicenter trial as active recruitment centers (i.e. University hospitals / Kantonsspitals in Aarau, Basel, Berne, Lucerne, Muri, Winterthur, Zofingen, Lausanne).

Additionally, we have two collaborating international sites in France and the United States, who will seek additional funding to join our efforts.

The Clinical Trial Unit (CTU) of the University of Basel will provide logistic and statistical support.

4. Design of the trial

4.1. Study design

This is an investigator-initiated, randomised controlled superiority trial with an open intervention comparing the effects of a nutritional intervention with a usual care control group.

4.2. Primary, secondary and safety endpoints

All patients will be assessed daily until hospital discharge and contacted after 30 and 180 days via telephone for a structured interview by blinded study nurses to assess primary and secondary endpoints.

The primary composite endpoint is combined adverse outcome within 30 days defined as

(a) All-cause mortality
(b) Admission to the intensive care unit from the medical ward
(c) Unplanned hospital readmission
(d) Major complications (defined according to previous trials[42]) as a new occurrence (being diagnosed after inclusion into the trial) of
   I. nosocomial infection or abscess requiring antibiotic treatment
   II. respiratory failure with need for invasive or non-invasive ventilation (continuous positive airway pressure, CPAP)
   III. major cardiovascular event (stroke, intracranial bleeding, cardiopulmonary arrest, myocardial infarction with and without invasive procedure) or pulmonary embolism
   IV. acute renal failure (defined by 2x increase of baseline creatinine or new requirement of dialysis do to volume overload or electrolyte disturbance)
   V. gastro-intestinal failure
(e) decline in functional status of 10% or more from admission to day 30 measured by the Barthel`s index[43] (Appendix 1). This index measures performance in activities of daily living and comprises two groups of items, one related to self-care (feeding, grooming, bathing, dressing, bowel and bladder care, and toilet use), the other related to mobility (ambulation, transfers, and stair climbing). The resulting score ranges from 100 to 0 with lower scores indicating more severe disability. For the purpose of this study, a significant decrease in functional outcome will be defined as a decrease in Barthel`s index by ≥10% from admission to day 30.

Secondary endpoints will be defined as follows:

(a) each single component of the primary endpoint at day 30
(b) each single component of the primary endpoint and the combined endpoint at short term, ie, at day 10 or hospital discharge whatever comes first
(c) each single component of the primary endpoint and the combined endpoint at long term term, ie, at day 180
(d) *short-term nutritional and functional outcomes* from inclusion to day 10 or hospital discharge (whatever comes first) including nutritional intake, improvement in muscle strength measured with handgrip strength[44], lean body mass, changes in undernutrition markers (pre-albumin, retinol-binding protein, body weight, BMI)];

(e) *hospital outcomes* measured at hospital discharge defined as total hospital LOS, discharge home vs. post-acute care facility, new decubital ulcer;

(f) Improvement in quality of life measured on admission and at 30-day and 180-day using the EuroQol Group 5-Dimension Self-Report Questionnaire (Appendix 3)[45] and selected items from the Functional Assessment Anorexia-Cancer Therapy [FAACT] questionnaire[46]),

<u>Safety endpoints including side effects</u> from nutritional therapy will be assessed daily until hospital discharge. A causal relationship with the trial will be rated by two study team members as unrelated / unlikely / possible / probable / definitely / not assessable. The following side effects were defined:

a) Adverse gastrointestinal effects (diarrhea, nausea, vomiting, abdominal pain)
b) Complications due to tube feeding or center catheter for parenteral nutrition,
c) Refeeding syndrome,
d) Liver or gall bladder dysfunction
e) Hyperglycemia (defined as glucose levels >12mmol/l or persistent levels >10mmol/l in patients without diabetes or well controlled diabetes)

4.3. Randomization and study procedures

If patients fulfill all inclusion criteria, with no exclusion criteria and provide informed consent, they will be randomized in a 1:1 fashion into the intervention group or the control group according to a pre-specified, computer-generated, web-based randomization scheme using the secuTrial© Software (managed and secured by the Clinical trial Unit of the University of Basel). The randomization will be stratified for the site and initial NRS (Appendix 2). In both study groups, all patients will be re-assessed daily by the nutritional specialist to re-evaluate nutritional intake and whether nutritional targets are met. If patients in the intervention group do not reach the nutritional targets (<75%), their nutritional strategy will be escalated according to the nutritional guidelines.

We will systematically collect blood samples at admission (day 0) and on inpatient days 2, 4, 6 and 8 for later batch measurement of nutritional markers and other biomarkers. If clinically indicated, there will be pre-prandial measurement of blood glucose levels 4 times daily and insulin treatment in all patients as part of usual clinical practice. There will also be regular measurement of nutritional factors in blood and possibly 24 hour urine to assess the effectiveness of nutritional therapy (see CRF for further details).

4.4. Initial patient assessment

After trial inclusion, each patient will receive a structured systematic medical and nutritional assessment by an experienced study-team member dietician according to the case report form (CRF) including:

- Socio-demographics (e.g., age, weight and height for calculation of body-mass index [BMI])

- If feasible, basal metabolic rate (BMR) calculated using indirect calorimetry; or if calorimetry is not feasible or tolerable, the Harris-Benedict formula will be used for estimation instead[40]
- Baseline lean body mass (estimated with body impedance analysis [BIA])[34]
- Baseline muscle strength (hand grip strength)[14]
- Baseline functional status (Barthel`s index)
- Medical diagnoses according to the ICD10-codes and nutritional diagnoses according to the IDNT reference manual (fourth edition)[41]

5. Selection of trial subjects

5.1. Recruitment

Upon hospital admission, consecutive adult (age ≥18 years) medical inpatients will be screened for undernutrition risk by the house nursing and /or physician staff, using the well-validated NRS, 2002 edition[38 39] , as shown in Appendix 2.

Based on this routine screening instrument, we will check daily for eligible patients using the hospital medical information system. Patients can be included in this trial within 24-48 hours of admission if they fulfill the following inclusion criteria

5.2. Inclusion criteria

Included will be all consecutive medical inpatients if they meet the following criteria

a) NRS ≥3 points
b) expected hospital LOS ≥5 days (as estimated by the treating physician team)
c) willingness to provide informed consent (see informed consent statement)

5.3. Exclusion criteria

Excluded will be patients if they meet the following criteria

a) initially admitted to critical care units (except intermediate care)
b) scheduled for surgery or in an immediate post-operative state
c) unable to ingest oral nutrition and thus need for enteral or parenteral nutrition
d) admitted with, or scheduled for, total parenteral nutrition or tube feeding
e) currently under nutritional therapy (defined by at least one visit with a dietician in the last month)
f) who are hospitalized because of anorexia nervosa
g) in terminal condition (end of life situation)
h) hospitalized due to acute pancreatitis
i) hospitalized due to acute liver failure
j) earlier inclusion into this trial

k) Cystic fibrosis

l) Patients after gastric bypass operations

m) Stem cell transplantation

n) Any contraindication against nutritional therapy (i.e., enteral and/or parenteral)

Figure 2: Study flow sheet

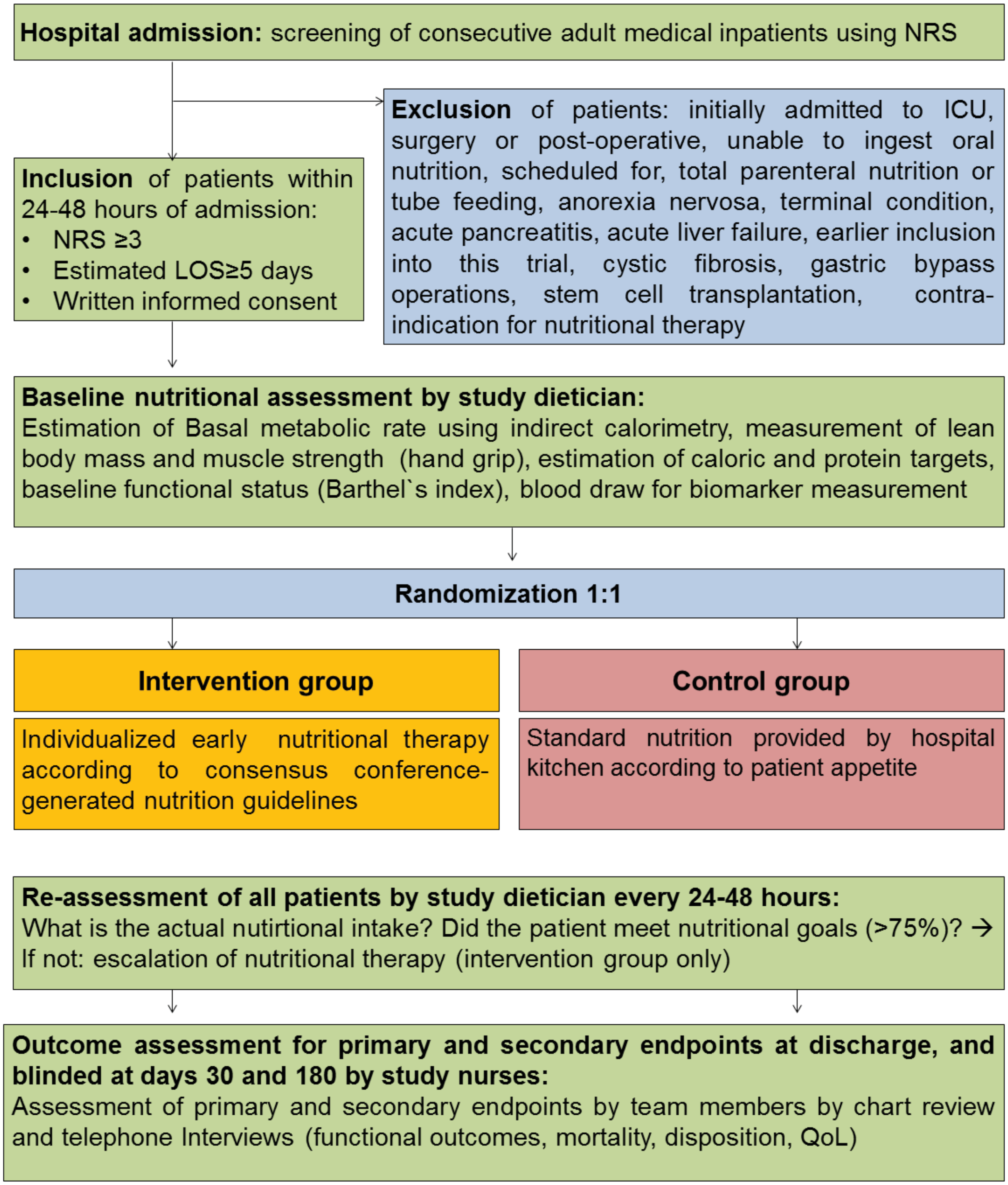

Figure 1: Nutritional strategy in the intervention group

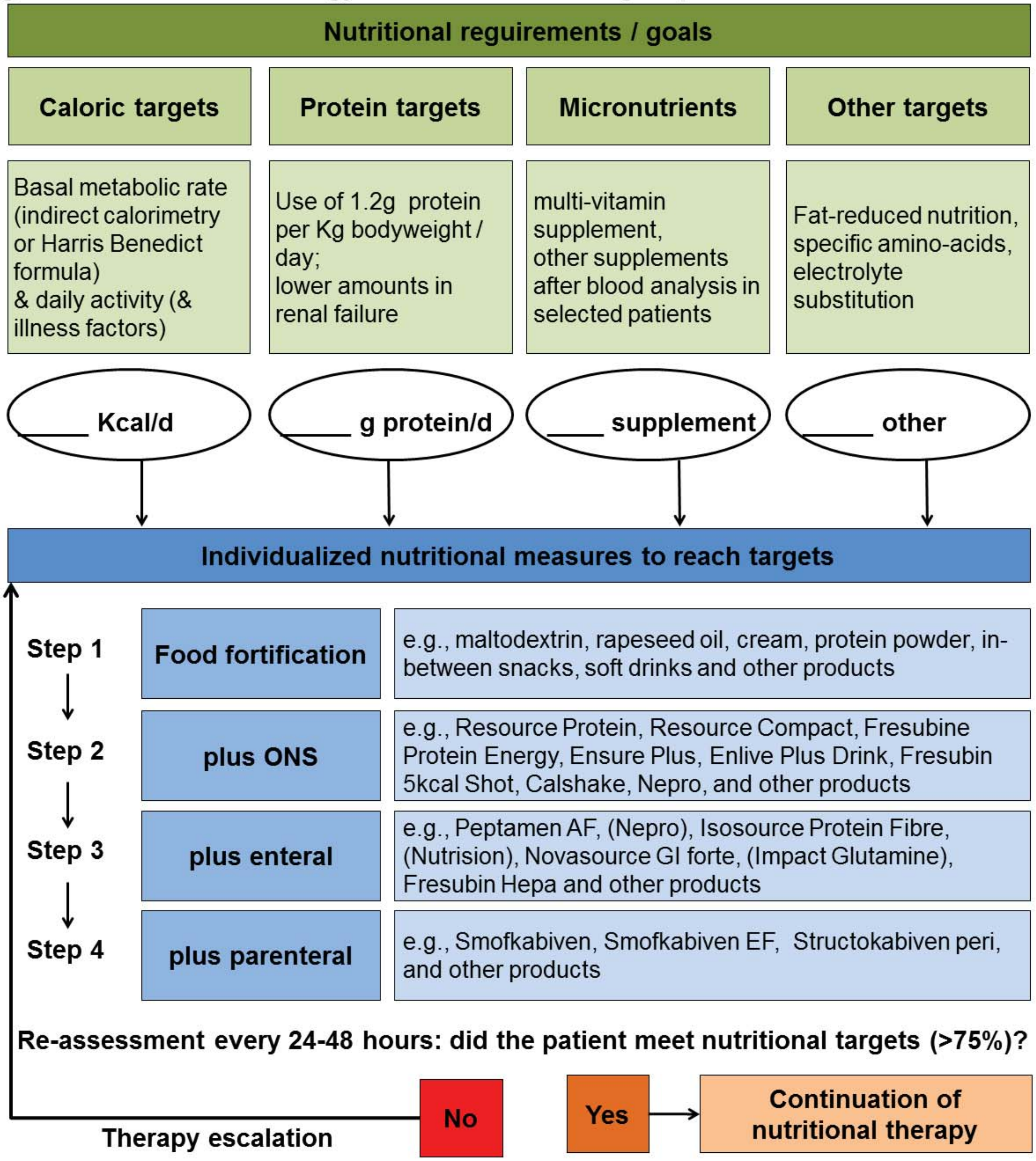

6. Assessment of effectiveness

Efficacy and response to treatment are evaluated by statistical analysis, comparing the intervention group to the control group regarding primary and secondary endpoints (see statistical analysis).

The analysis will be performed together with the clinical trial unit (CTU) of the University of Basel by using the collected data from the study database which will be similar for all participating study centers.

6.1. Intervention

For the purpose of this study, we have developed nutritional guidelines by consensus and adapted to current guidelines (e.g., ESPEN, ASPEN) [21-34] (*figure 1 and Appendix 4*). Guidelines specify a reinforced nutritional therapy strategy to cover nutritional requirements, focusing on nutritional targets based on the specific nutritional diagnoses defined by the IDNT[41]. The nutritional guidelines may vary according to important medical diagnoses (i.e. renal failure). They specify not only nutritional targets, but also escalation of the route (i.e. food fortification, oral, enteral, parenteral) if targets cannot be achieved (≤75%) every 24-48 hours.

Nutritional goals will be assessed daily in patients in the intervention group (*Figure 2*). In control patients, we will use conventional nutrition according to the ability and desire of the patient to eat, using standard care food provided by the hospital kitchen ("appetite-guided").

Nutritional therapy may be started in control patients at any time, if new swallowing disorders develop or if patients need to be prepared for surgical interventions. Similarly, nutritional therapy may be stopped in case patients in the intervention group become terminal or develop a condition where nutritional therapy is contraindicated (e.g., intestinal perforation).Thus, in both groups the nutritional therapy protocol may be overruled after discussion with the PI and the involved study coordinators.

6.2. Blinding

Patients and caregivers will not be blinded to the intervention (i.e., no placebo-controlled intervention) as this would be neither practical not feasible given the different nutritional treatment options available. However, we will have blinded outcome assessment at day 30 and day 180 in regard to primary and secondary outcomes.

7. Assessment of Safety

7.1. Risks of the study

We consider the risks of this study to be minimal, for the following reasons:

- Blood Loss: 7.5ml of EDTA-plasma and 7.5ml of serum as well as one additional tube (7.5ml) for determination of genetic markers will be additionally drawn on admission. All other blood draws are part of clinical routine
- Whether or not nutritional therapy has a benefit in acutely ill medical inpatients remains unclear. Different smaller trials have not been able to demonstrate large improvements in patient outcomes in regard to mortality and/or morbidity.
- Risks of nutritional therapy include refeeding syndrome and risk associated with enteral and parenteral nutrition. We will do a careful monitoring for refeeding including daily clinical visits and routine blood work for electrolyte dysbalance. In case of refeeding syndrome, we will lower the amount of nutritional therapy (50%) and also treat with B-complex vitamins. We expect that only a minority of patients will receive enteral and parenteral nutrition (around 10%) and in these patients benefits may outweigh risks.

8. Statistics

8.1. Analysis population

The primary analysis population is the full analysis set, which, following intention-to treat (ITT) principles, includes all randomized patients. Every effort will be made to minimize the number of patients lost to follow-up.

8.2. Statistical approach

Detailed methodology for summaries and statistical analyses of the data collected in this study will be documented in a statistical analysis plan. The statistical analysis plan will be finalized before database closure.

Briefly, a consort diagram will be reported including all screened patients, patients lost to follow up and patients included in the final analyses. Primary and secondary endpoints will be compared between trial arms in the overall ITT population and within predefined subgroups as discussed below. All outcomes will be analyzed in an uncorrected manner as well as jointly corrected for the main risk factors (Barthel`s index at baseline, study center and initial NRS categories). For our primary analysis, we will compare the two arms with a chi-square test and we will also estimate effect size with a logistic regression model reporting unadjusted and adjusted odds ratios (OR) and corresponding 95% confidence intervals (CIs).

We will do different predefined subgroup analyses by including interaction terms in the regression models to test for effect modification by important baseline factors. Specifically, we will look at patients age (<60, 60-75, >75 years), gender, risk for undernutrition stratified according to initial NRS (3, 4, >4 points), BMI (<20, 20-25, >25-30, >30), main medical diagnosis (systemic infection, heart failure, acute renal failure, gastro-intestinal disease, tumor), comorbidities (diabetes, chronic renal failure).

For the analysis of functional outcome (defined by Barthel`s index) we will use a non-parametric Wilcoxon rank sum test to compare Barthel`s index at day 30 between randomization arms. However, to adjust for additional variables, we will also fit a multivariate linear model including Barthel`s index at baseline, study center and initial NRS to estimate effect size and 95% confidence intervals (95%CIs). If needed, data will be transformed as appropriate (e.g., log transformation). Moreover, we will assess the progression of Barthel`s index over time (i.e. admission, day 8, day 30) using a linear mixed model approach to account for the repeated measurements per patient.

A similar approach will be used for all other secondary endpoints where we will use chi-square tests / logistic regression analysis for binary outcomes, Wilcoxon rank sum test / linear models for continuous outcomes and log-rank test / cox regression for time-to-event data as appropriate.

8.3. Sample Size considerations

This study is designed to show superiority of nutritional therapy compared to “appetite-guided” standard care regarding the composite primary endpoint. Our hypothesis is that early nutritional therapy will reduce mortality and morbidity within a follow up period of 30 days after the index hospitalization. From preliminary observational data collected during the TRIAGE pilot study[47], we estimate that 40% of the target patient population (NRS ≥3 points and LOS ≥5days) will reach the primary endpoint within 30 days (10% mortality, 5% ICU admission from the hospital ward, 15% complications, 10% functional decline with 10% reaching more than 1 endpoint). We hypothesize that our nutritional intervention will decrease this risk by an absolute number of 6% (relative decrease of 15%), i.e., from 40% to 34%. For a comparison of two independent binomial proportions using Pearson's Chi-square statistic with a Chi-square approximation with a two-sided significance level of 0.05, a sample size of 1'359 per group achieves a power of at least 0.9 when the proportions are 0.4 and 0.34.

Thus, a total number of 2000-3000 patients will provide the study with a power of 78-90% to detect a 15% relative decrease in the risk to reach the primary endpoint. The table below shows sample sizes for different assumptions regarding effectiveness of our intervention and power.

Sample size considerations in regard to the primary endpoint

Frequency of 1° EP intervention group	Frequency of 1° EP control group	Difference control/intervention groups	Power	Patients per group
0.4	0.36	-10%	0.8	2311
0.4	0.36	-10%	0.85	2643
0.4	0.36	-10%	0.9	3093
0.4	0.34	-15%	0.8	1016
0.4	0.34	-15%	0.85	1162
0.4	0.34	-15%	0.9	1359
0.4	0.32	-20%	0.8	564
0.4	0.32	-20%	0.85	645
0.4	0.32	-20%	0.9	755

8.4. Pilot phase and trial duration:

Prior to start of this complex trial, we will do a 3-6 month pilot to study feasibility. During this time period we will validate all trial related materials (e.g. case report forms, patient and health care worker information sheets) and test feasibility of our intervention. Based on these results we may make last-minute protocol enhancements. Thereafter, we include study centers in a stepwise fashion for a maximal total enrollment period of 3 years.

9. Trial-specific preventive measures and duties

9.1. Trial monitoring

In accordance with applicable regulations and good clinical practice (GCP), monitors will periodically contact the different sites, including on-site visits. The extent, nature and frequency of on-site visits will be based on enrolment rate, the quality of the documents provided by the site, consistency of follow-up of the patients according to this protocol.

During these contacts, the monitor will check and assess the progress of the trial, review trial data collected, conduct source document verification and identify any issues and address their resolution.

This will be done in order to verify that the data are authentic, accurate, and complete, that safety and rights of subjects are being protected and that the trial is conducted in accordance with the currently approved protocol (and any amendments), GCP, and all applicable regulatory requirements.

The investigator agrees to allow the monitor direct access to all relevant documents and to allocate his/her time and the time of his/her staff to the monitor to discuss findings and any relevant issues.

In addition to contacts during the trial, the monitor will also contact the site prior to the start of the trial to discuss the protocol and data collection procedures with site personnel.

9.2. Adverse outcome

An adverse event in a subject is defined as any untoward occurrence of any unfavorable and unintended clinically relevant medical sign, symptom, or disease temporally associated with the study which must not necessarily have a causal relationship with the study procedure. All adverse events of category III or more according to Common Terminology Criteria for Adverse Events (CTCAE) v4.0 will be recorded. Adverse events will be monitored for every subject participating in the study and attributed to the study procedures / design by the study investigators. Any serious unexpected adverse event will be categorized into the following categories:

Unrelated

There is no evidence of any causal relationship.

Unlikely

There is little evidence to suggest there is a causal relationship (e.g. the event did not occur within a reasonable time after administration of the IMP). There is another reasonable explanation for the event (e.g. the participant's clinical condition, other concomitant treatment).

Possible

There is some evidence to suggest a causal relationship (e.g. because the event occurs within a reasonable time after administration of the IMP). However, the influence of other factors may have contributed to the event (e.g. the participant's clinical condition, other concomitant treatments).

Probable

There is evidence to suggest a causal relationship and the influence of other factors is unlikely.

Definitely

There is clear evidence to suggest a causal relationship and other possible contributing factors can be ruled out.

Not assessable

There is insufficient or incomplete evidence to make a clinical judgment of the causal relationship.

The following grade levels will be used in defining the severity of adverse events:

Adverse Event (AE):

Any untoward medical occurrence in a clinical trial subject which does not necessarily have a causal relationship with this treatment. An AE can therefore be any unfavorable and unintended sign (including an abnormal laboratory finding), symptom, or disease temporally associated with the trial, whether or not considered related to the trial.

Adverse Reaction (AR):

All untoward and unintended responses to the intervention judged by either the reporting investigator or the sponsor as having a reasonable causal relationship to the trial. The expression reasonable causal relationship means to convey in general that there is evidence or argument to suggest a causal relationship.

Unexpected Adverse Reaction (UAR):

An AR, the nature or severity of which is not consistent with the applicable product information (e.g. investigator's brochure for an unapproved investigational product or summary of product characteristics (SmPC) for an authorized product). When the outcome of the adverse reaction is not consistent with the applicable product information this adverse reaction should be considered as unexpected. Side effects documented in the IB or SmPC which occur in a more severe form than anticipated are also considered as being unexpected.

Serious Adverse Event (SAE) or Serious Adverse Reaction:

Any untoward medical occurrence or effect that at any dose results in death, is life-threatening, requires hospitalization, or prolongation of existing hospitalization, results in persistent or significant disability or incapacity or is a congenital anomaly or birth defect. In this context, the term life threatening refers to an event in which the trial participant was at immediate risk of death at the time of the event; it does not refer to an event, which might have caused death if it were more severe.

Suspected Unexpected Serious Adverse Reaction (SUSAR):

Any suspected adverse reaction related to an IMP that is both unexpected and serious.

All adverse events must be followed until resolution, until the condition stabilizes, until the event is otherwise explained, or the subject is lost to follow-up. The investigator is responsible to ensure that follow-up includes any supplemental investigations that may be indicated to completely evaluate the nature and/or causality of the adverse event. This may include additional laboratory tests or

investigations, histo-pathological examinations, or consultation with other health care professionals, and reports.

Reporting of Serious Adverse Events

All SAEs and SUSARs must be reported to the sponsor within 24 - 48 hours of knowledge. For documentation a trial specific SAE report form provided by the sponsor must be filled in. Collection of complete information concerning SAEs is extremely important. If not available at time of the initial report, detailed information about SAEs should be given in the follow-up report together with all relevant documentation e.g. laboratory and hospital discharge reports.

The sponsor will review these cases. In case of SUSAR, these cases will be reported to the DSMB. SAE will be reported annually to the ethics committee and to the reporting authorities; life threatening SUSAR or SUSAR resulting in death will be reported to the ethics committee and to the reporting authorities (Switzerland: Swissmedic, Safety of Medicines Division; vigilance@swissmedic.ch) within 7 days, all other SUSAR within 15 days of knowledge, according to local law.

The study coordinating centre may request that the investigators perform or arrange for the conduct of supplemental measurements and/or evaluations. If a subject dies during participation in the trial, the study coordinating centre should be provided with a copy of any post-mortem findings, including histo-pathology.

10. Duties on the part of the investigator

The investigators and institutions will permit study related monitoring visits, audits, IEC reviews, and regulatory inspections, and provide direct access to all source data.

Source data are all information, original records of clinical findings, observations, or other activities in this clinical trial necessary for the reconstruction and evaluation of the trial.

In accordance with applicable regulations and good clinical practice (GCP), monitors will periodically contact the site, including on-site visits. The extent, nature and frequency of on-site visits will be based on enrolment rate, the quality of the documents provided by the site, consistency of follow-up of the patients according to this protocol.

During these contacts, the monitor will check and assess the progress of the trial, review trial data collected, conduct source document verification and identify any issues and address their resolution.

This will be done in order to verify that the data are authentic, accurate, and complete, that safety and rights of subjects are being protected and that the trial is conducted in accordance with the currently approved protocol (and any amendments), GCP, and all applicable regulatory requirements.

The investigator agrees to allow the monitor direct access to all relevant documents and to allocate his/her time and the time of his/her staff to the monitor to discuss findings and any relevant issues.

In addition to contacts during the trial, the monitor will also contact the site prior to the start of the trial to discuss the protocol and data collection procedures with site personnel.

11. Ethical considerations

The study will be approved by the ethics committees of the participating hospitals.

This study will be conducted in compliance with the protocol approved by the IEC, and according to ICH-Good Clinical Practice standards. No deviation from the protocol will be implemented without prior review and approval of the IEC except where it may be necessary to eliminate an immediate hazard to a research subject. In such case, the deviation will be reported to the IEC as soon as possible.All enrolled patients will be asked to give written informed consent. In patients, in which “informed consent” is not feasible due to dementia or their acute medical condition, patients’ next to kin can sign an assent form to state the presumptive will of the patient. In case, next of kin are not readily available, a treating physician – who must not be involved in the study – have to certify that there are no objections for inclusion in the study from his point of view. Only after these informed consent procedures the patient can be included in the study.

Importantly, despite strong associations between malnutrition and adverse clinical outcome, we believe that is ethically acceptable that the control group receive usual care treatment because there is uncertainty about the effectiveness and safety of nutritional therapy in this patient population. This important subject has been discussed among national experts in the field (i.e., collaborators) who all agree to this practice. This is also in accordance with a recent Swiss consensus ethical statement pointing out that “intake of standard food and fluids is a basic right of any patients”, yet any sort of nutritional therapy must be viewed as a therapeutic measure and must therefore fulfill all criteria for such including proof of clinical effectiveness, safety and cost-effectiveness)[37]. For our patient population, such proofs is still missing and the main aim of this trial.

12. Quality control and assurance: description of measures

The study will be submitted to the institutional review boards of all involved hospitals and will be registered in the Current Controlled Trials Database. Written informed consent will be obtained from all included patients or their legal representatives.

At its discretion, the study coordinating centre may conduct a quality assurance audit of this trial. If such an audit occurs, the investigator agrees to allow the auditor direct access to all relevant documents and to allocate his/her time and the time of his/her staff to the auditor to discuss findings and any relevant issues. A guideline for audit is available at the study coordinating centre.

In addition, regulatory agencies may conduct a regulatory inspection of this trial. If such an inspection occurs, the investigator agrees to allow the inspector direct access to all relevant documents and to allocate his/her time and the time of his/her staff to the inspector to discuss findings and any relevant issues.

12.1. Quality assurance and data/record keeping

The Sponsor is responsible for implementing and maintaining quality assurance and quality control systems with written SOPs and Working Instructions, at all sites. The PI is responsible for proper training of all involved study personnel.

All study related documents (essential documents and site documents) are archived at the local sites or electronically (electronic CRFs) for 10 years. On the stored CRFs, participants cannot be identified by name and birth date. Appropriate coded identification, will be used.t

All blood samples will be stored at the Kantonsspital Aarau for a maximum duration of 10 years and will then be destroyed.

13. Ancillary projects

13.1. Physio-pathological mechanisms of action of nutritional therapy

Purpose: To understand the physio-pathological mechanisms of action explaining how nutritional therapy exerts its effects on disease recovery in specific patient populations and illnesses. The large sample size and the diversity in medical diagnosis and comorbidities will offer the unique opportunity to study distinct effects of nutritional therapy.

General approach: Using patient data generated in the RCT, we will first look at how effects of nutritional therapy vary depending on age (quartiles), gender, medical illnesses (systemic infection, heart failure, renal failure, gastro-intestinal disease, tumor), BMI (<20, 20-25, >25-30, >30) and acuity (acute vs. chronic disease courses). We will also look at subgroups defined by admission nutritional / endocrine markers (e.g. initial pre-albumin, stress markers, inflammation blood biomarkers). In a second step, we will focus on specific patient populations to assess whether nutritional therapy has an effect on disease course. Specifically, we will focus on patients with systemic infections to study effects of nutritional therapy on the inflammatory response. We will look at biomarkers measured in blood samples collected from patients on admission and during follow up (inpatient days 0, 1, 3, 5 and 7). We will look at levels of inflammatory/infection biomarkers such as C-reactive protein (CRP) and PCT and stress markers such as cortisol and copeptin levels as surrogates for disease severity, recovery and medical outcome, respectively.

Statistical approach: Detailed methodology for the statistical analyses will be documented in a statistical analysis plan. To investigate whether differential treatment effects exist in patient subgroups as defined above, we will include interaction terms into the statistical model and check for effect modification (see details in RCT section, statistical approach). For subgroups defined by biomarker levels, we will divide those levels into quartiles to check for robustness of results. All analyses will be predefined based on biological plausibility to avoid multiple testing. We will further define a derivation sample and a validation sample (1:1) for these analyses for reproducibility of results and decreased risk of chance findings. We will use a linear mixed model approach to assess the effect of nutrition on serial biomarker levels over the course of hospitalization.

13.2. Cost-effectiveness analysis of nutritional therapy

Purpose: to perform a cost-effectiveness calculation for early nutritional therapy in medical inpatients at risk for undernutrition including cost-effectiveness and cost-utility analyses. These analyses will be from the health-care payers' perspective using intervention data generated in the RCT.

General approach: The total cost calculation will include nutritional costs (including nutritional products and estimated time of dieticians spent with patients) and all medical costs within 30 days after study inclusion. The total cost calculation also will include all hospitalisations after index hospitalisation. We

will derive estimates of the real costs using REKOLE a comprehensive accounting algorithm used by Swiss hospitals to internally compute provider costs. Cost-effectiveness will be expressed as the incremental cost-effectiveness ratio. Non-parametric bootstrapping will be used to estimate 95%CIs for differences in incremental costs and effects. We will further calculate the probability value that nutritional therapy is cost-effective for a range of different willingness to pay values. We will define confidence intervals for the relevant cost difference. We will assume equivalence if the difference in total direct costs will lie between the equivalence margins. Non-inferiority of costs will be demonstrated if the upper limit of the confidence interval for cost differences between the two strategies will be smaller than the cost difference. In a second step we will perform a joint analysis of effects and costs in a cost-utility analysis by analysing the cost per quality adjusted life years (QALY) gained. We will present an overall analysis, and different subgroup analyses as defined in the RCT. Specifically, we will look at patients age (<60, 60-75, >75 years), gender, risk for undernutrition stratified according to initial NRS (3, 4, >4 points), main medical diagnosis (systemic infection, heart failure, acute renal failure, gastro-intestinal disease, tumor), comorbidities (diabetes, chronic renal failure), LOS (<8 days, ≥8 days) and BMI (<20, 20-25, >25-30, >30).

Presentation of results: To assess the shape of the joint sampling distribution of costs and effects we will present the joint density distribution of incremental costs and effects on the cost-effectiveness plane. We will further estimate the incremental net monetary benefit and the uncertainty surrounding this estimate. We will visualise the uncertainty surrounding the cost-effectiveness estimates by deriving the cost-effectiveness acceptability curves. These curves provide estimates of the probability that the intervention is cost-effective for a range of different willingness to pay values (*Figure 3*)[48].

Figure 3: Visualization of cost-effectiveness

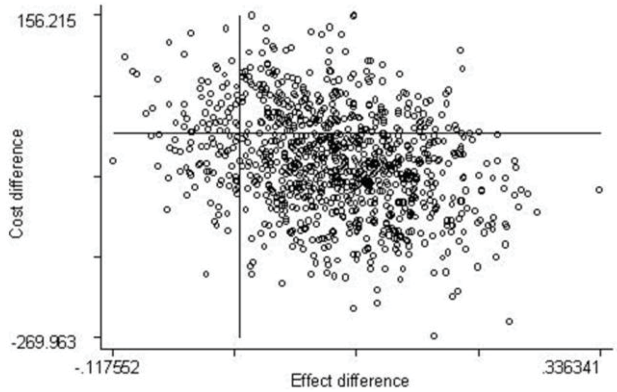

Because acceptability curves focus on the incremental cost effectiveness ratio as a continuous measure in relation to the amount a policymaker is willing to pay to gain one unit of effect, acceptability curves have no meaningful interpretation in economic noninferiority research. We will therefore define cost and effect margins (based on the cost difference for one day of hospitalisation and based on an effect difference of 2 points for QoL in the EuroQol Score) for a non-inferiority scenario in case we cannot show superiority in costs and comparable effects of the two investigated management strategies under investigation. Finally the uncertainty around the treatment decision will be expressed in terms of the total expected value of perfect information[49].

Outlook: No prospective trial data are available for incremental cost-effectiveness of nutritional therapy in a large unselected population of medical inpatients outside critical care. This analysis will provide

conclusive evidence about expected costs and benefits of nutritional therapy in different types of medical patients for more rational decision-making of healthcare providers.

13.3. Impact of nutritional support on glucose metabolism and insulin regimens

Hyperglycemia and insulin resistance are common in acutely ill medical patients, even in those without a history of diabetes mellitus[50]. We and others found strong associations of hyperglycemia and adverse outcomes of patients with systemic infection hospitalized in the non-critical care setting[50-52]. Similar data have been found in other patient populations, such as those suffering from stroke[53]. Preclinical data suggest a profound influence of hyperglycemia on immune function. Hyperglycemia interacts in vitro with components of the innate immune system, such as chemotaxis, phagocytosis, and macrophage activation[54-56]. In animal models of diabetes, hyperglycemia was associated with decreased bacterial clearance and higher infection-related mortality[57]. Generally, hyperglycemia was considered to reflect severity of illness. Yet, provision of nutritional therapy may further increase blood glucose levels and – in the absence of adequate insulin therapy - may thereby contribute to the deleterious effects of hyperglycemia. While the effects of nutritional therapy on glucose metabolism have been well studied in the critical care setting[58], similar data in medical inpatients are largely lacking.

To close this gap, we will study the influence of nutritional therapy on glucose homeostasis and insulin management as a possible explanation for the higher rates of complication and infection of patients with high caloric intake. Specifically, we will routinely collect information about glucose control, i.e., 4-times-daily glucose measurements and insulin dose if applicable in all included patients. We hypothesize that hyperglycemia on admission and during inpatient treatment is associated with an adverse disease course, as evidenced by more pronounced inflammatory and stress responses and worse clinical outcomes. To test this hypothesis, we will first investigate the association of initial blood glucose concentrations with clinical outcomes including LOS, disease-specific complications, and mortality. Based on the initial glucose concentration, patients will be priori classified as having normal levels (reference group, blood glucose <7mmol/L), moderate hyperglycemia (blood glucose 7-11mmol/L) or severe hyperglycemia (blood glucose >11mmol/L) based on the classification in a recent publication[59]. In patients with systemic infection, we will used blood levels of CRP and PCT and the white blood cells count at admission and on days 3, 5 and 7 to reflect the inflammatory response. In a second step, we will compare glucose metabolism between randomization arms and investigate whether glucose control with insulin may reduce any harmful effects exerted by hyperglycemia.

This ancillary project will shed light on the important interplay of nutritional therapy and glucose metabolism, and may help to generate preliminary data on the optimal use of insulin in this situation.

13.4. Biosampling for later detection of genetic modulators

The term “personalized medicine” relates to the observation that not all patients show the same response to medical therapies. For example, while some patients may show a marked benefit from nutritional therapy, other patients may have no benefit or may even suffer harm from that intervention. Whether or not a patient benefits from nutritional therapy may relate to illness-specific factors (e.g., comorbidities, acute vs. chronic course) or patient-specific factors (e.g., age and gender). Additionally, we hypothesize that genetic signatures exist which will help in identifying patients who may or may not benefit from nutritional therapy.

A number of studies have found an association between specific DNA polymorphisms and the amount, function, or both of the gene products produced in response to pathogenic stimuli. In case of patients with systemic infection and sepsis, for example, these include genes that code for a variety of pattern recognition receptors[64-66] and polymorphisms have been implicated in mortality. For example, an A to G substitution at the –308 position in the TNF-α promoter was associated with elevated TNF-α expression in vitro and in vivo, and was shown to confer a 4-fold increased mortality in one study of septic shock.[64] The association between gene polymorphisms and medical outcomes awaits large-scale validation. However, there is strong support for the inclusion of genotyping as an important consideration when designing clinical trials such as EFFORT[65-67].

To study the impact of genotyping on nutritional therapy, we will perform biosampling in all patients upon their enrolment into this trial (using a separate informed consent). Specifically we will collect one PAXgene Blood RNA tubes (5.0 ml) and one PAXgene Blood DNA tube (8.5 ml) for later mRNA, miRNA and DNA analysis using cutting-edge techniques, such as next generation sequencing (NGS) and microarray analysis in collaboration with Prof. A. Lauber (Department of Endocrinology, University of Fribourg) and the Functional Genomic Center Zurich, with whom we have on ongoing collaboration. This approach proved to be successful as demonstrated by the existence of several “nutrigenomics” networks, aiming to eventually lead to evidence-based nutritional interventions for restoring health and fitness and for preventing diet-dependent diseases (reviewed in[68]). The financial support for this project is not a part of the present proposal.

13.5. Importance of nurse-sensitive factors

Nutritional therapy appears to be a complex intervention that acts on different levels. While EFFORT focuses primarily on the efficacy of the nutritional strategy, other elements, such as nurse-sensitive factors, may be of particular importance in the successful implementation of such a strategy in “real life.” To further shed light on this important topic, we will in collaboration with Prof. S. De Geest of the Institute of Nursing Science, University of Basel, develop a protocol to study the importance of various nurse-sensitive factors (e.g., nurse workload, nurse staffing, nursing staff attitudes towards nutritional therapy) on our intervention. For this purpose, we will at all study sites collect caregiver-level data and conduct interviews with nursing staff. We will then investigate whether such factors help account for differences

in the magnitude of patient response to our intervention. Specifically, we will test for effect modification within our regression model using a hierarchical approach. A more detailed proposal will be developed in the later preparation phase of the trial.

14. Schedule and milestones

EFFORT includes several research aims which will consecutively be accomplished as demonstrated below. For the prospective trial, we will do a 3-6 month pilot-phase with step-wise inclusion of study centers followed by a 2-year (maximal 3 year) active patient recruitment period. Thereafter, we have dedicated 18 month to complete the 6 month long term follow-up of all patients, and finish all primary and secondary aims and ancillary projects.

Objectives	Jan - Jun 2014	Jul - Dec 2014	Jan - Jun 2015	Jul - Dec 2015	Jan - Jun 2016	Jul - Dec 2016	Jan - Jun 2017	Jul - Dec 2017	Jan - Jun 2018
**Main trial**									
Finalization of protocol, ethical approval	■	■							
Pilot phase, stepwise inclusion of centers		■							
Active patients enrollment in all centers			■	■	■	■			
Database finalization and 6-month follow-up			■	■	■	■	■		
Data analysis and manuscript preparation							■	■	■
Manuscript publication								■	■
**Physio-pathological effects**									
Data analysis							■	■	■
Manuscript preparation and publication								■	■
**Cost-effectiveness analysis**									
Data analysis							■	■	■
Manuscript preparation and publication								■	■

15. Potential limitations

We are aware of several potential limitations to the successful completion of this trial. First, inclusion of 3000 patients over a 2-year time frame is ambitious. However, based on our experience from previous multicenter randomized trials, our well-established multicenter research network, the conceptual and logistical support from the University of Basel CTU, and the large total population of patients potentially eligible for this trial who are hospitalized at the study centers, we are convinced that the trial is feasible. Pilot data from a current observational cohort study have shown that the University Department of Medicine at the Kantonsspital Aarau by itself has about 8,000 medical hospitalizations per year, among which about 1,500 – 2,000 patients have an NRS≥3 points and a LOS≥5 days[47] and would thus be enrollment candidates. Also, before active recruitment begins, we have planned a pilot feasibility period to validate all trial-related material as well as the intervention. Last-minute protocol enhancements to address “real-world issues” (if any) would be possible.

As a second limitation, there is no blinding of patients or caregivers regarding the randomization arm, which might introduce bias. However, outcome assessment at day 30, including for the primary endpoint and most secondary endpoints will be blinded. Due to the variety of nutritional options to reach the nutritional goals, we felt that a placebo control group would be neither feasible, nor ethical.

Third, there is potential for control group “contamination”, i.e., if the caregiver staff feels obliged to motivate patients in the control group to increase their food consumption. A complex intervention such as nutritional therapy must be implemented at different levels and by the full care team in the hospital. While dieticians recommend individualized strategies for patients, the physician staff need to support the strategy and motivate the patient, and, most importantly, the nursing team is key in everyday application of the strategy, i.e., actively encouraging, and if needed, feeding the patient. It will thus be important to continuously educate the caregiver staff about the intention of this trial, and the potential risks of nutritional therapy which have not yet been well-studied in the population in question. We will also study caregiver characteristics (e.g., nurse–sensitive factors potentially affecting outcome, such as nursing staff work load) to detect any impact of these variables on our intervention (ancillary project).

Fourth, it is expected that not all patients in the intervention group reach their caloric and protein goals and some may refuse enteral and parenteral nutrition. However, as a pragmatic, trial we are most interested in the effects of nutritional therapy in “real life” using a state-of-the-art algorithm. Still, we will document actual nutritional intake and later study the effect of compliance on our intervention.

16. Importance and impact of the proposed project

Nutrition is essential for survival and function in health and disease. Despite its centrality to hospital practice, nutritional therapy has not been well-studied and high-quality evidence of efficacy, safety and cost-effectiveness in acutely-ill medical inpatients outside critical care is lacking. These gaps and ambiguities in the literature and recent evidence from critically ill patients[4, 7] suggesting potential harmful effects of nutritional therapy are of concern.

By integrating international expertise, a strong research infrastructure, comprehensive meta-analysis, and an original, large-scale, interventional RCT, EFFORT aims to establish an evidence-based standard for nutritional therapy in medical inpatients. Additionally, using a physio-pathological mechanistic approach, EFFORT will increase basic understanding on how nutrition affects acute disease and vice versa. Further, by incorporating pharmaco-economic research, EFFORT will elucidate the indications in which nutritional therapy, which because of its widespread application currently is associated with substantial costs, is cost-effective and those in which it is not. By generating these data, EFFORT will facilitate more efficient healthcare resource distribution.

In the centerpiece of EFFORT, we shall test this strategy in the largest-yet nutritional RCT outside critical care to provide definitive evidence about expected benefits and harms of this intervention. Additionally,

we shall capitalize on the EFFORT RCT to study physio-pathological mechanisms associated with the interplay of nutrition and disease, and to measure the value of nutritional therapy.

This pragmatic comparative effectiveness research project thereby will improve the quality, effectiveness, safety and efficiency of nutritional therapy and basic understanding of the relationship between nutrition and illness. Data acquired through EFFORT will help healthcare professionals and payers worldwide to make better-informed decisions regarding care of frail, elderly and polymorbid individuals with acute illness, who represent a large and growing patient population worldwide, and one that accounts for a major share of medical resource consumption. We thus expect that the results of all aims of the EFFORT project will be widely, directly and rapidly applied – and indeed, will contribute to a new standard of care -- in hospital clinical practice worldwide.

17. REFERENCES

1. Kubrak C, Jensen L: Malnutrition in acute care patients: a narrative review. *Int J Nurs Stud* 2007, 44(6):1036-1054.
2. Kuhlmann MK, Levin NW: Potential interplay between nutrition and inflammation in dialysis patients. *Contrib Nephrol* 2008, 161:76-82.
3. Oner-Iyidogan Y, Gurdol F, Kocak H, Oner P, Cetinalp-Demircan P, Caliskan Y, Kocak T, Turkmen A: Appetite-regulating hormones in chronic kidney disease patients. *J Ren Nutr* 2011, 21(4):316-321.
4. Casaer MP, Hermans G, Wilmer A, Van den Berghe G: Impact of early parenteral nutrition completing enteral nutrition in adult critically ill patients (EPaNIC trial): a study protocol and statistical analysis plan for a randomized controlled trial. *Trials* 2011, 12:21.
5. Villet S, Chiolero RL, Bollmann MD, Revelly JP, Cayeux RNM, Delarue J, Berger MM: Negative impact of hypocaloric feeding and energy balance on clinical outcome in ICU patients. *Clin Nutr* 2005, 24(4):502-509.
6. Casaer MP, Mesotten D, Hermans G, Wouters PJ, Schetz M, Meyfroidt G, Van Cromphaut S, Ingels C, Meersseman P, Muller J, Vlasselaers D, Debaveye Y, Desmet L, Dubois J, Van Assche A, Vanderheyden S, Wilmer A, Van den Berghe G: Early versus late parenteral nutrition in critically ill adults. *N Engl J Med* 2011, 365(6):506-517.
7. Heyland D, Muscedere J, Wischmeyer PE, Cook D, Jones G, Albert M, Elke G, Berger MM, Day AG, Canadian Critical Care Trials G: A randomized trial of glutamine and antioxidants in critically ill patients. *N Engl J Med* 2013, 368(16):1489-1497.
8. Heidegger CP, Berger MM, Graf S, Zingg W, Darmon P, Costanza MC, Thibault R, Pichard C: Optimisation of energy provision with supplemental parenteral nutrition in critically ill patients: a randomised controlled clinical trial. *Lancet* 2013, 381(9864):385-393.
9. Koretz RL, Avenell A, Lipman TO, Braunschweig CL, Milne AC: Does enteral nutrition affect clinical outcome? A systematic review of the randomized trials. *Am J Gastroenterol* 2007, 102(2):412-429; quiz 468.
10. Milne AC, Potter J, Vivanti A, Avenell A: Protein and energy supplementation in elderly people at risk from malnutrition. *Cochrane Database Syst Rev* 2009(2):CD003288.
11. Somanchi M, Tao X, Mullin GE: The facilitated early enteral and dietary management effectiveness trial in hospitalized patients with malnutrition. *JPEN J Parenter Enteral Nutr* 2011, 35(2):209-216.
12. Starke J, Schneider H, Alteheld B, Stehle P, Meier R: Short-term individual nutritional care as part of routine clinical setting improves outcome and quality of life in malnourished medical patients. *Clin Nutr* 2011, 30(2):194-201.
13. Rufenacht U, Ruhlin M, Wegmann M, Imoberdorf R, Ballmer PE: Nutritional counseling improves quality of life and nutrient intake in hospitalized undernourished patients. *Nutrition* 2010, 26(1):53-60.
14. Norman K, Kirchner H, Freudenreich M, Ockenga J, Lochs H, Pirlich M: Three month intervention with protein and energy rich supplements improve muscle function and quality of life in malnourished patients with non-neoplastic gastrointestinal disease--a randomized controlled trial. *Clin Nutr* 2008, 27(1):48-56.
15. Gariballa S, Forster S: Dietary supplementation and quality of life of older patients: a randomized, double-blind, placebo-controlled trial. *J Am Geriatr Soc* 2007, 55(12):2030-2034.
16. Gariballa S, Forster S, Walters S, Powers H: A randomized, double-blind, placebo-controlled trial of nutritional supplementation during acute illness. *Am J Med* 2006, 119(8):693-699.
17. Johansen N, Kondrup J, Plum LM, Bak L, Norregaard P, Bunch E, Baernthsen H, Andersen JR, Larsen IH, Martinsen A: Effect of nutritional support on clinical outcome in patients at nutritional risk. *Clin Nutr* 2004, 23(4):539-550.
18. Hickson M, Bulpitt C, Nunes M, Peters R, Cooke J, Nicholl C, Frost G: Does additional feeding support provided by health care assistants improve nutritional status and outcome in acutely ill older in-patients?--a randomised control trial. *Clin Nutr* 2004, 23(1):69-77.
19. Schetz M, Casaer MP, Van den Berghe G: Does artificial nutrition improve outcome of critical illness? *Crit Care* 2013, 17(1):302.
20. Vincent JL, Preiser JC: When should we add parenteral to enteral nutrition? *Lancet* 2013, 381(9864):354-355.
21. Volkert D, Berner YN, Berry E, Cederholm T, Coti Bertrand P, Milne A, Palmblad J, Schneider S, Sobotka L, Stanga Z, Lenzen-Grossimlinghaus R, Krys U, Pirlich M, Herbst B, Schutz T, Schroer W, Weinrebe W,

Ockenga J, Lochs H: ESPEN Guidelines on Enteral Nutrition: Geriatrics. *Clinical nutrition* 2006, 25(2):330-360.

22. Vanek VW, Matarese LE, Robinson M, Sacks GS, Young LS, Kochevar M: A.S.P.E.N. position paper: parenteral nutrition glutamine supplementation. *Nutr Clin Pract* 2011, 26(4):479-494.
23. Vanek VW, Borum P, Buchman A, Fessler TA, Howard L, Jeejeebhoy K, Kochevar M, Shenkin A, Valentine CJ: A.S.P.E.N. position paper: recommendations for changes in commercially available parenteral multivitamin and multi-trace element products. *Nutr Clin Pract* 2012, 27(4):440-491.
24. Sobotka L, Schneider SM, Berner YN, Cederholm T, Krznaric Z, Shenkin A, Stanga Z, Toigo G, Vandewoude M, Volkert D: ESPEN Guidelines on Parenteral Nutrition: geriatrics. *Clinical nutrition* 2009, 28(4):461-466.
25. Plauth M, Cabre E, Riggio O, Assis-Camilo M, Pirlich M, Kondrup J, Ferenci P, Holm E, Vom Dahl S, Muller MJ, Nolte W: ESPEN Guidelines on Enteral Nutrition: Liver disease. *Clinical nutrition* 2006, 25(2):285-294.
26. Mueller C, Compher C, Ellen DM: A.S.P.E.N. clinical guidelines: Nutrition screening, assessment, and intervention in adults. *JPEN J Parenter Enteral Nutr* 2011, 35(1):16-24.
27. McMahon MM, Nystrom E, Braunschweig C, Miles J, Compher C: A.S.P.E.N. clinical guidelines: nutrition support of adult patients with hyperglycemia. *JPEN J Parenter Enteral Nutr* 2013, 37(1):23-36.
28. Choban P, Dickerson R, Malone A, Worthington P, Compher C: A.S.P.E.N. Clinical Guidelines: Nutrition Support of Hospitalized Adult Patients With Obesity. *JPEN J Parenter Enteral Nutr* 2013.
29. Cano NJ, Aparicio M, Brunori G, Carrero JJ, Cianciaruso B, Fiaccadori E, Lindholm B, Teplan V, Fouque D, Guarnieri G: ESPEN Guidelines on Parenteral Nutrition: adult renal failure. *Clinical nutrition* 2009, 28(4):401-414.
30. Brown RO, Compher C: A.S.P.E.N. clinical guidelines: nutrition support in adult acute and chronic renal failure. *JPEN J Parenter Enteral Nutr* 2010, 34(4):366-377.
31. August DA, Huhmann MB: A.S.P.E.N. clinical guidelines: nutrition support therapy during adult anticancer treatment and in hematopoietic cell transplantation. *JPEN J Parenter Enteral Nutr* 2009, 33(5):472-500.
32. Arends J, Bodoky G, Bozzetti F, Fearon K, Muscaritoli M, Selga G, van Bokhorst-de van der Schueren MA, von Meyenfeldt M, Zurcher G, Fietkau R, Aulbert E, Frick B, Holm M, Kneba M, Mestrom HJ, Zander A: ESPEN Guidelines on Enteral Nutrition: Non-surgical oncology. *Clinical nutrition* 2006, 25(2):245-259.
33. Volkert D, Berner YN, Berry E, Cederholm T, Coti Bertrand P, Milne A, Palmblad J, Schneider S, Sobotka L, Stanga Z, Dgem, Lenzen-Grossimlinghaus R, Krys U, Pirlich M, Herbst B, Schutz T, Schroer W, Weinrebe W, Ockenga J, Lochs H, Espen: ESPEN Guidelines on Enteral Nutrition: Geriatrics. *Clin Nutr* 2006, 25(2):330-360.
34. Maisonneuve N, Genton L, Karsegard VL, Kyle UG, Dupertuis YM, Pichard C: [Role of impedance measurement in nutritional screening]. *Rev Med Suisse Romande* 2004, 124(10):611-615.
35. Tinetti ME, Studenski SA: Comparative effectiveness research and patients with multiple chronic conditions. *N Engl J Med* 2011, 364(26):2478-2481.
36. Van Spall HG, Toren A, Kiss A, Fowler RA: Eligibility criteria of randomized controlled trials published in high-impact general medical journals: a systematic sampling review. *JAMA* 2007, 297(11):1233-1240.
37. Baumann-Hölzle R, Imoberdorf R, Koblet K, Ballmer P, Rühlin M, Bau R, Büchs U, Cabiallavetta B, Ehrensperger K, Fendt Y, Haller A, Meier-Allmendinger D, Stuber P, Winzeler M, Kündig A: Ernährungsautonomie – ethisches Grundsatzpapier zur Ernährung der Patientinnen und Patienten im Akutspital. *Schweizerische Ärztezeitung* 2006, 87:33.
38. Kondrup J, Johansen N, Plum LM, Bak L, Larsen IH, Martinsen A, Andersen JR, Baernthsen H, Bunch E, Lauesen N: Incidence of nutritional risk and causes of inadequate nutritional care in hospitals. *Clin Nutr* 2002, 21(6):461-468.
39. Kondrup J, Allison SP, Elia M, Vellas B, Plauth M, Educational, Clinical Practice Committee ESoP, Enteral N: ESPEN guidelines for nutrition screening 2002. *Clin Nutr* 2003, 22(4):415-421.
40. Gaillard C, Alix E, Salle A, Berrut G, Ritz P: Energy requirements in frail elderly people: a review of the literature. *Clin Nutr* 2007, 26(1):16-24.
41. Atkins M, Basualdo-Hammond C, Hotson B, Dietitians of C: Canadian perspectives on the nutrition care process and international dietetics and nutrition terminology. *Can J Diet Pract Res* 2010, 71(2):e18-20.
42. Buzby GP, Knox LS, Crosby LO, Eisenberg JM, Haakenson CM, McNeal GE, Page CP, Peterson OL, Reinhardt GF, Williford WO: Study protocol: a randomized clinical trial of total parenteral nutrition in malnourished surgical patients. *Am J Clin Nutr* 1988, 47(2 Suppl):366-381.
43. Mahoney FI, Barthel DW: Functional Evaluation: The Barthel Index. *Md State Med J* 1965, 14:61-65.

44. Hermans G, Clerckx B, Vanhullebusch T, Segers J, Vanpee G, Robbeets C, Casaer MP, Wouters P, Gosselink R, Van Den Berghe G: Interobserver agreement of Medical Research Council sum-score and handgrip strength in the intensive care unit. *Muscle Nerve* 2012, 45(1):18-25.

45. Brooks R: EuroQol: the current state of play. *Health Policy* 1996, 37(1):53-72.

46. Ribaudo JM, Cella D, Hahn EA, Lloyd SR, Tchekmedyian NS, Von Roenn J, Leslie WT: Re-validation and shortening of the Functional Assessment of Anorexia/Cachexia Therapy (FAACT) questionnaire. *Qual Life Res* 2000, 9(10):1137-1146.

47. Schuetz P, Hausfater P, Amin D, Haubitz S, Fassler L, Grolimund E, Kutz A, Schild U, Caldara Z, Regez K, Zhydkov A, Kahles T, Nedeltchev K, von Felten S, De Geest S, Conca A, Schafer-Keller P, Huber A, Bargetzi M, Buergi U, Sauvin G, Perrig-Chiello P, Reutlinger B, Mueller B: Optimizing triage and hospitalization in adult general medical emergency patients: the triage project. *BMC Emerg Med* 2013, 13(1):12.

48. Fenwick E, O'Brien BJ, Briggs A: Cost-effectiveness acceptability curves--facts, fallacies and frequently asked questions. *Health Econ* 2004, 13(5):405-415.

49. Ades AE, Lu G, Claxton K: Expected value of sample information calculations in medical decision modeling. *Med Decis Making* 2004, 24(2):207-227.

50. Schuetz P, Jones AE, Howell MD, Trzeciak S, Ngo L, Younger JG, Aird W, Shapiro NI: Diabetes is not associated with increased mortality in emergency department patients with sepsis. *Ann Emerg Med* 2011, 58(5):438-444.

51. Schuetz P, Kennedy M, Lucas JM, Howell MD, Aird WC, Yealy DM, Shapiro NI: Initial management of septic patients with hyperglycemia in the noncritical care inpatient setting. *Am J Med* 2012, 125(7):670-678.

52. Schuetz P, Yano K, Sorasaki M, Ngo L, St Hilaire M, Lucas JM, Aird W, Shapiro NI: Influence of diabetes on endothelial cell response during sepsis. *Diabetologia* 2011, 54(5):996-1003.

53. Bolk J, van der Ploeg T, Cornel JH, Arnold AE, Sepers J, Umans VA: Impaired glucose metabolism predicts mortality after a myocardial infarction. *Int J Cardiol* 2001, 79(2-3):207-214.

54. Delamaire M, Maugendre D, Moreno M, Le Goff MC, Allannic H, Genetet B: Impaired leucocyte functions in diabetic patients. *Diabet Med* 1997, 14(1):29-34.

55. Alexiewicz JM, Kumar D, Smogorzewski M, Klin M, Massry SG: Polymorphonuclear leukocytes in non-insulin-dependent diabetes mellitus: abnormalities in metabolism and function. *Ann Intern Med* 1995, 123(12):919-924.

56. Schuetz P, Castro P, Shapiro NI: Diabetes and sepsis: preclinical findings and clinical relevance. *Diabetes Care* 2011, 34(3):771-778.

57. Edwards MS, Fuselier PA: Enhanced susceptibility of mice with streptozotocin-induced diabetes to type II group B streptococcal infection. *Infect Immun* 1983, 39(2):580-585.

58. Debaveye Y, Van den Berghe G: Risks and benefits of nutritional support during critical illness. *Annu Rev Nutr* 2006, 26:513-538.

59. Lepper PM, Ott S, Nuesch E, von Eynatten M, Schumann C, Pletz MW, Mealing NM, Welte T, Bauer TT, Suttorp N, Juni P, Bals R, Rohde G, German Community Acquired Pneumonia Competence N: Serum glucose levels for predicting death in patients admitted to hospital for community acquired pneumonia: prospective cohort study. *BMJ* 2012, 344:e3397.

60. Engel JH, Siewerdt F, Jackson R, Akobundu U, Wait C, Sahyoun N: Hardiness, depression, and emotional well-being and their association with appetite in older adults. *J Am Geriatr Soc* 2011, 59(3):482-487.

61. Vizza J, Neatrour DM, Felton PM, Ellsworth DL: Improvement in psychosocial functioning during an intensive cardiovascular lifestyle modification program. *J Cardiopulm Rehabil Prev* 2007, 27(6):376-383; quiz 384-375.

62. Kanazawa Y, Nakao T, Ohya Y, Shimomitsu T: Association of socio-psychological factors with the effects of low protein diet for the prevention of the progression of chronic renal failure. *Intern Med* 2006, 45(4):199-206.

63. Morley JE: Pathophysiology of the anorexia of aging. *Curr Opin Clin Nutr Metab Care* 2013, 16(1):27-32.

64. Mira JP, Cariou A, Grall F, Delclaux C, Losser MR, Heshmati F, Cheval C, Monchi M, Teboul JL, Riche F, Leleu G, Arbibe L, Mignon A, Delpech M, Dhainaut JF: Association of TNF2, a TNF-alpha promoter polymorphism, with septic shock susceptibility and mortality: a multicenter study. *Jama* 1999, 282(6):561-568.

65. Lin MT, Albertson TE: Genomic polymorphisms in sepsis. *Crit Care Med* 2004, 32(2):569-579.

66. Holmes CL, Russell JA, Walley KR: Genetic polymorphisms in sepsis and septic shock: role in prognosis and potential for therapy. *Chest* 2003, 124(3):1103-1115.

67. Angus DC, Burgner D, Wunderink R, Mira JP, Gerlach H, Wiedermann CJ, Vincent JL: The PIRO concept: P is for predisposition. *Crit Care* 2003, 7(3):248-251.

68. Afman L, Muller M: Nutrigenomics: from molecular nutrition to prevention of disease. *J Am Diet Assoc* 2006, 106(4):569-576.

APPENDIX

APPENIDX 1. Barthel index (German version)

Patientendaten

Aktivitäten des täglichen Lebens (ADL), Barthel-Index

Datum:

1. Essen	Punkte	
Unabhängig, benutzt Geschirr und Besteck	10	
Braucht Hilfe, z.B. beim Schneiden	5	
Total hilfsbedürftig	0	
**2. Baden**		
Badet oder duscht ohne Hilfe	5	
Badet oder duscht mit Hilfe	0	
**3. Waschen**		
Wäscht Gesicht, kämmt, rasiert bzw. schminkt sich, putzt Zähne	5	
Braucht Hilfe	0	
**4. An- und Auskleiden**		
Unabhängig, inkl. Schuhe anziehen	10	
Hilfsbedürftig, kleidet sich teilweise selbst an/aus	5	
Total hilfsbedürftig	0	
**5. Stuhlkontrolle**		
Kontinent	10	
Teilweise inkontinent	5	
Inkontinent	0	
**6. Urinkontrolle**		
Kontinent	10	
Teilweise inkontinent	5	
Inkontinent	0	
**7. Toilettenbenutzung**		
Unabhängig bei Benutzung der Toilette/des Nachtstuhls	10	
Braucht Hilfe für z.B. Gleichgewicht, Kleidung aus-/anziehen, Toilettenpapier	5	
Kann nicht auf Toilette/Nachtstuhl	0	
**8. Bett-/(Roll-)Stuhltransfer**		
Unabhängig (gilt auch für Rollstuhlfahrer)	15	
Minimale Assistenz oder Supervision	10	
Kann sitzen, braucht für den Transfer jedoch Hilfe	5	
Bettlägerig	0	
**9. Bewegung**		
Unabhängiges Gehen (auch mit Gehhilfe) für mindestens 50 m	15	
Mindestens 50 m Gehen, jedoch mit Unterstützung	10	
Für Rollstuhlfahrer: unabhängig für mindestens 50 m	5	
Kann sich nicht mindestens 50 m fortbewegen	0	
**10. Treppensteigen**		
Unabhängig (auch mit Gehhilfe)	10	
Braucht Hilfe oder Supervision	5	
Kann nicht Treppensteigen	0	
**Gesamtpunktzahl (max. 100)**		

APPENIDX 2. Nutritional risk screening (NRS, 2002)

Screening auf Mangelernährung im Krankenhaus

Nutritional Risk Screening (NRS 2002)

nach Kondrup J et al., Clinical Nutrition 2003; 22: 415-421

Empfohlen von der Europäischen Gesellschaft für Klinische Ernährung und Stoffwechsel (ESPEN)

Vorscreening:

- Ist der Body Mass Index < 20,5 kg/m² ? ☐ ja ☐ nein
- Hat der Patient in den vergangenen 3 Monaten an Gewicht verloren? ☐ ja ☐ nein
- War die Nahrungszufuhr in der vergangenen Woche vermindert? ☐ ja ☐ nein
- Ist der Patient schwer erkrankt? (z.B. Intensivtherapie) ☐ ja ☐ nein

⇒ Wird eine dieser Fragen mit „Ja" beantwortet, wird mit dem Hauptscreening fortgefahren

⇒ Werden alle Fragen mit „Nein" beantwortet, wird der Patient wöchentlich neu gescreent.

⇒ Wenn für den Patienten z.B. eine große Operation geplant ist, sollte ein präventiver Ernährungsplan verfolgt werden, um dem assoziierte Risiko vorzubeugen.

Hauptscreening:

Störung des Ernährungszustands	Punkte
**Keine**	0
**Mild**<br>Gewichtsverlust > 5%/ 3 Mo. oder Nahrungszufuhr < 50-75% des Bedarfes in der vergangenen Woche	1
**Mäßig**<br>Gewichtsverlust > 5%/ 2 Mo. oder BMI 18,5-20,5 kg/m²<br>und reduzierter Allgemeinzustand (AZ)<br>oder Nahrungszufuhr 25-50% des Bedarfes in der vergangenen Woche	2
**Schwer**<br>Gewichtsverlust> 5% /1 Mo. (>15% / 3 Mo.)<br>oder BMI <18,5 kg/m² und reduzierter Allgemeinzustand oder Nahrungszufuhr 0-25% des Bedarfes in der vergangenen Woche	3

+

Krankheitsschwere	Punkte
**Keine**	0
**Mild**<br>z.B. Schenkelhalsfraktur, chronische Erkrankungen besonders mit Komplikationen: Leberzirrhose, chronisch obstruktive Lungenerkrankung, chronische Hämodialyse, Diabetes, Krebsleiden	1
**Mäßig**<br>z.B. große Bauchchirurgie, Schlaganfall, schwere Pneumonie, hämatologische Krebserkrankung	2
**Schwer**<br>z.B. Kopfverletzung, Knochenmarktransplantation, intensivpflichtige Patienten (APACHE-II >10)	3

+ 1 Punkt, wenn Alter ≥ 70 Jahre

≥ 3 Punkte	Ernährungsrisiko liegt vor, Erstellung eines Ernährungsplanes
< 3 Punkte	wöchentlich wiederholtes Screening. Wenn für den Patienten z.B. eine große Operation geplant ist, sollte ein präventiver Ernährungsplan verfolgt werden, um das assoziierte Risiko zu vermeiden

T. Schütz, L. Valentini, M. Plauth. Screening auf Mangelernährung nach den ESPEN-Leitlinien 2002. Aktuel Ernaehr Med 2005; 30: 99-103.

APPENIDX 3. Quality of life (EQ 5D)

Bitte geben Sie an, welche Aussagen Ihren heutigen Gesundheitszustand am besten beschreiben, indem Sie ein Kreuz in ein Kästchen jeder Gruppe machen.

Beweglichkeit/Mobilität

Ich habe keine Probleme herumzugehen ❑
Ich habe einige Probleme herumzugehen ❑
Ich bin ans Bett gebunden ❑

Für sich selbst sorgen

Ich habe keine Probleme, für mich selbst zu sorgen ❑
Ich habe einige Probleme, mich selbst zu waschen oder mich anzuziehen ❑
Ich bin nicht in der Lage, mich selbst zu waschen oder anzuziehen ❑

Allgemeine Tätigkeiten *(z.B. Arbeit, Studium, Hausarbeit, Familien- oder Freizeitaktivitäten)*

Ich habe keine Probleme, meinen alltäglichen Tätigkeiten nachzugehen ❑
Ich habe einige Probleme, meinen alltäglichen Tätigkeiten nachzugehen ❑
Ich bin nicht in der Lage, meinen alltäglichen Tätigkeiten nachzugehen ❑

Schmerzen/körperliche Beschwerden

Ich habe keine Schmerzen oder Beschwerden ❑
Ich habe mässige Schmerzen oder Beschwerden ❑
Ich habe extreme Schmerzen oder Beschwerden ❑

Angst/Niedergeschlagenheit

Ich bin nicht ängstlich oder deprimiert ❑
Ich bin mässig ängstlich oder deprimiert ❑
Ich bin extrem ängstlich oder deprimiert ❑

Um Sie bei der Einschätzung, wie gut oder wie schlecht Ihr Gesundheitszustand ist, zu unterstützen, haben wir eine Skala gezeichnet, ähnlich einem Thermometer. Der best denkbare Gesundheitszustand ist mit 100 gekennzeichnet, der schlechteste mit 0.

Wir möchten Sie nun bitten, auf dieser Skala zu kennzeichnen, wie gut oder schlecht Ihrer Ansicht nach Ihr persönlicher Gesundheitszustand heute ist. Bitte verbinden Sie dazu den unten stehenden Kasten mit dem Punkt auf der Skala, der Ihren heutigen Gesundheitszustand am besten wiedergibt.

Ihr

heutiger Gesundheitszustand

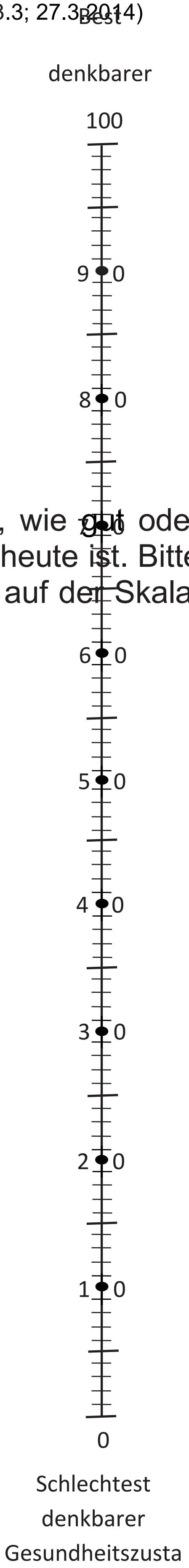

APPENIDX 4. Nutritional guidelines (German version)

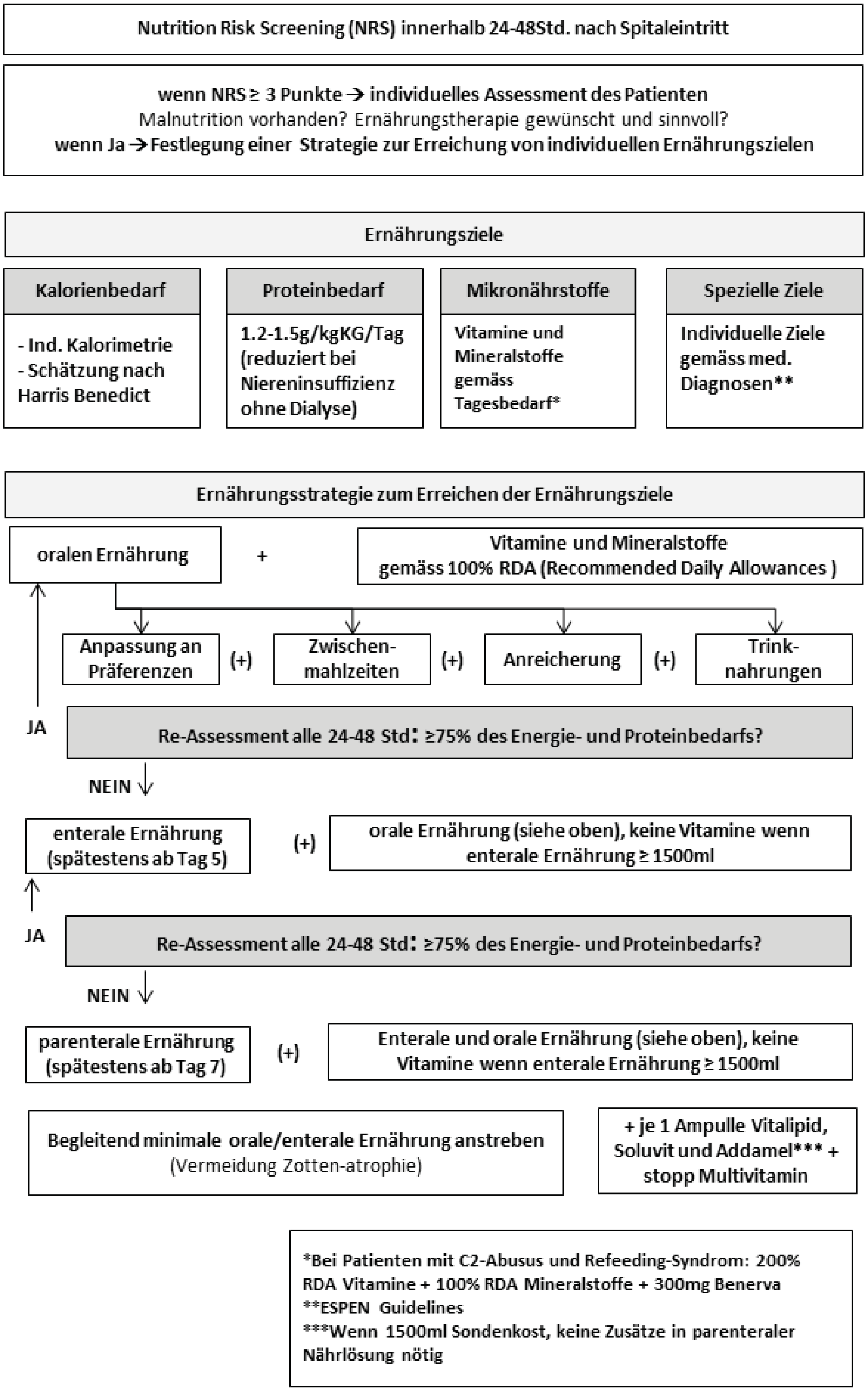

Supplement: Supplement 1. — Trial Protocol [file jamanetwopen-3-e200663-s001.pdf]
