# Supplementary material for: Association of Baseline Inflammation With Effectiveness of Nutritional Support Among Patients With Disease-Related Malnutrition: A Secondary Analysis of a Randomized Clinical Trial
Source: JAMA Netw Open. 2020 Mar 10;3(3):e200663. doi: 10.1001/jamanetworkopen.2020.0663 (PMC7064875; doi:10.1001/jamanetworkopen.2020.0663)
Supplement: Supplement 2. — eTable. Baseline Characteristics According to Randomization Arm Overall and Stratified by CRP Status [file jamanetwopen-3-e200663-s002.pdf]

Supplementary Online Content

Merker M, Felder M, Gueissaz L, et al. Association of baseline inflammation with effectiveness of nutritional support among patients with disease-related malnutrition: a secondary analysis of a randomized clinical trial. *JAMA Netw Open*. 2020;3(3):e200663. doi:10.1001/jamanetworkopen.2020.0663

eTable. Baseline Characteristics According to Randomization Arm Overall and Stratified by CRP Status

This supplementary material has been provided by the authors to give readers additional information about their work.

© 2020 Merker M et al. *JAMA Network Open.*

eTable. Baseline Characteristics According to Randomization Arm Overall and Stratified by CRP Status

	**CRP value (<10mg/l)**			**CRP (10-100mg/l)**			**CRP (>100mg/l)**		
**Characteristics**	**Control group**	**Intervention group**	**p-value**	**Control group**	**Intervention group**	**p-value**	**Control group**	**Intervention group**	**p-value**
**n (%)**	281	252		429	465		262	261	
**Demographic characteristics**									
Age, years (median, IQR)	74.0 (61.0, 82.0)	75.0 (63.0, 84.0)	0.14	77.0 (68.0, 84.0)	75.0 (65.0, 82.0)	0.099	75.0 (68.0, 81.0)	74.0 (65.0, 81.0)	0.24
Male sex	134 (47.7%)	116 (46.0%)		240 (55.9%)	250 (53.8%)		143 (54.6%)	142 (54.4%)	
BMI, kg/m^2 (median, IQR)	23.0 (20.0, 27.0)	23.0 (20.0, 27.0)	0.93	24.0 (21.0, 28.0)	24.0 (21.0, 28.0)	0.33	25.0 (22.0, 28.0)	24.0 (21.0, 28.0)	0.49
BMI WHO									
< 18.5 kg/m2	39 (13.9%)	33 (13.1%)	0.95	42 (9.8%)	25 (5.4%)	0.036	14 (5.4%)	20 (7.7%)	0.54
18.5-25 kg/m2	152 (54.1%)	136 (54.0%)		217 (50.6%)	256 (55.4%)		129 (49.4%)	127 (49.0%)	
>25 kg/m2	90 (32.0%)	83 (32.9%)		170 (39.6%)	181 (39.2%)		118 (45.2%)	112 (43.2%)	
NRS 2002									
3 points	107 (38.1%)	91 (36.1%)	0.93	137 (31.9%)	157 (33.8%)	0.93	54 (20.6%)	52 (19.9%)	0.61
4 points	110 (39.1%)	101 (40.1%)		161 (37.5%)	167 (35.9%)		99 (37.8%)	113 (43.3%)	
5 points	57 (20.3%)	55 (21.8%)		111 (25.9%)	121 (26.0%)		83 (31.7%)	72 (27.6%)	
≥6 points	7 (2.5%)	5 (2.0%)		20 (4.7%)	20 (4.3%)		26 (9.9%)	24 (9.2%)	
**Main diagnosis**									
Cardiovascular disease	41 (14.6%)	31 (12.3%)	0.31	61 (14.2%)	53 (11.4%)	0.32	6 (2.3%)	5 (1.9%)	0.092
Infectious disease	19 (6.8%)	24 (9.5%)		121 (28.2%)	114 (24.5%)		165 (63.0%)	149 (57.1%)	
Metabolic disorder	16 (5.7%)	19 (7.5%)		14 (3.3%)	10 (2.2%)		1 (0.4%)	0 (0.0%)	
Gastrointestinal disease	23 (8.2%)	29 (11.5%)		35 (8.2%)	51 (11.0%)		5 (1.9%)	13 (5.0%)	
Renal disease	9 (3.2%)	9 (3.6%)		20 (4.7%)	18 (3.9%)		4 (1.5%)	6 (2.3%)	
Cancer	48 (17.1%)	41 (16.3%)		72 (16.8%)	102 (21.9%)		45 (17.2%)	52 (19.9%)	
Pulmonary disease	18 (6.4%)	13 (5.2%)		35 (8.2%)	27 (5.8%)		17 (6.5%)	7 (2.7%)	
Neurological disorder	40 (14.2%)	24 (9.5%)		10 (2.3%)	12 (2.6%)		0 (0.0%)	5 (1.9%)	
Frailty	49 (17.4%)	35 (13.9%)		35 (8.2%)	45 (9.7%)		10 (3.8%)	14 (5.4%)	
Other	7 (2.5%)	7 (2.8%)		11 (2.6%)	14 (3.0%)		5 (1.9%)	6 (2.3%)	
Comorbidities									
Coronary heart disease	80 (28.5%)	77 (30.6%)	0.60	132 (30.8%)	122 (26.2%)	0.13	55 (21.0%)	73 (28.0%)	0.064
Congestive heart failure	44 (15.7%)	42 (16.7%)	0.75	86 (20.0%)	94 (20.2%)	0.95	39 (14.9%)	36 (13.8%)	0.72
Hypertension	146 (52.0%)	141 (56.0%)	0.36	240 (55.9%)	248 (53.3%)	0.43	141 (53.8%)	146 (55.9%)	0.63
Cerebrovascular Disease	26 (9.3%)	23 (9.1%)	0.96	39 (9.1%)	30 (6.5%)	0.14	19 (7.3%)	21 (8.0%)	0.73
Peripheral arterial disease	26 (9.3%)	24 (9.5%)	0.91	52 (12.1%)	35 (7.5%)	0.021	19 (7.3%)	19 (7.3%)	0.99
Chronic kidney disease	74 (26.3%)	73 (29.0%)	0.50	152 (35.4%)	156 (33.5%)	0.55	80 (30.5%)	83 (31.8%)	0.75
Diabetes	48 (17.1%)	58 (23.0%)	0.087	100 (23.3%)	94 (20.2%)	0.26	55 (21.0%)	52 (19.9%)	0.76
COPD	41 (14.6%)	34 (13.5%)	0.72	73 (17.0%)	70 (15.1%)	0.42	35 (13.4%)	38 (14.6%)	0.69
Dementia	12 (4.3%)	12 (4.8%)	0.78	16 (3.7%)	15 (3.2%)	0.68	6 (2.3%)	11 (4.2%)	0.21
Malignant disease	77 (27.4%)	66 (26.2%)	0.75	147 (34.3%)	162 (34.8%)	0.86	96 (36.6%)	99 (37.9%)	0.76
**Clinical findings**									
Barthel index	95 (80, 100)	90 (75, 100)	0.15	90 (70, 100)	90 (70, 95)	0.49	85 (65, 95)	85 (70, 95)	0.36
median (IQR)	144 (51.2%)	121 (48.0%)	0.46	170 (39.6%)	182 (39.1%)	0.88	96 (36.6%)	94 (36.0%)	0.88
functional impairment (<90 points)	137 (48.8%)	131 (52.0%)		259 (60.4%)	283 (60.9%)		166 (63.4%)	167 (64.0%)	
**CRP levels**									
Admission CRP levels									
median (IQR)	4.0 (3.0, 5.3)	3.6 (3.0, 5.4)	0.74	35.0 (19, 64)	34 (18, 62)	0.80	174 (131, 227)	170 (140, 230)	0.77
mean (SD)	4.3 (2.2)	4.3 (2.3)	0.90	42.1 (26.4)	41.8 (26.7)	0.88	189.6 (72.6)	193.4 (77.4)	0.57
**Nutritional Intake during the trial**									
Mean daily protein intake	52.7 (20.5)	59.2 (23.0)	<0.001	44.5 (21.7)	57.0 (22.8)	<0.001	44.7 (19.5)	54.6 (23.5)	<0.001
Mean daily calorie intake	1362 (524)	1571 (601)	<0.001	1150 (511)	1502 (591)	<0.001	1138 (499)	1432 (606)	<0.001

© 2020 Merker M et al. *JAMA Network Open.*
