# Supplementary material for: Association of Baseline Inflammation With Effectiveness of Nutritional Support Among Patients With Disease-Related Malnutrition: A Secondary Analysis of a Randomized Clinical Trial
Source: JAMA Netw Open. 2020 Mar 10;3(3):e200663. doi: 10.1001/jamanetworkopen.2020.0663 (PMC7064875; doi:10.1001/jamanetworkopen.2020.0663)
Supplement: Supplement 3. — Data Sharing Statement [file jamanetwopen-3-e200663-s003.pdf]

Data Sharing Statement

Merker. Association of Baseline Inflammation With Effectiveness of Nutritional Support Among Patients With Disease-Related Malnutrition. *JAMA Netw Open*. Published March 10, 2020. 10.1001/jamanetworkopen.2020.0663

Data
Data available: Yes
Data types: Deidentified participant data
How to access data: contact the corresponding author
When available: With publication

Supporting Documents
Document types: None

Additional Information
Who can access the data: NA
Types of analyses: NA
Mechanisms of data availability: NA
Any additional restrictions: NA
